# Stratifying and predicting progression to acute liver failure during the early phase of acute liver injury

**DOI:** 10.1093/pnasnexus/pgaf004

**Published:** 2025-02-06

**Authors:** Raiki Yoshimura, Masatake Tanaka, Miho Kurokawa, Naotoshi Nakamura, Takeshi Goya, Koji Imoto, Motoyuki Kohjima, Katsuhito Fujiu, Shingo Iwami, Yoshihiro Ogawa

**Affiliations:** Interdisciplinary Biology Laboratory (iBLab), Division of Natural Science, Graduate School of Science, Nagoya University, Aichi 464-8602, Japan; Department of Medicine and Bioregulatory Science, Graduate School of Medical Sciences, Kyushu University, Fukuoka 812-8582, Japan; Department of Medicine and Bioregulatory Science, Graduate School of Medical Sciences, Kyushu University, Fukuoka 812-8582, Japan; Interdisciplinary Biology Laboratory (iBLab), Division of Natural Science, Graduate School of Science, Nagoya University, Aichi 464-8602, Japan; Department of Medicine and Bioregulatory Science, Graduate School of Medical Sciences, Kyushu University, Fukuoka 812-8582, Japan; Department of Medicine and Bioregulatory Science, Graduate School of Medical Sciences, Kyushu University, Fukuoka 812-8582, Japan; Department of Gastroenterology, NHO Kyushu Medical Center, Fukuoka 810-8563, Japan; Department of Cardiovascular Medicine, Graduate School of Medicine, The University of Tokyo, Tokyo 113-0033, Japan; Interdisciplinary Biology Laboratory (iBLab), Division of Natural Science, Graduate School of Science, Nagoya University, Aichi 464-8602, Japan; Institute of Mathematics for Industry, Kyushu University, Fukuoka 819-0395, Japan; Institute for the Advanced Study of Human Biology (ASHBi), Kyoto University, Kyoto 606-8501, Japan; Interdisciplinary Theoretical and Mathematical Sciences Program (iTHEMS), RIKEN, Saitama 351-0198, Japan; NEXT-Ganken Program, Japanese Foundation for Cancer Research (JFCR), Tokyo 135-8550, Japan; International Research Center for Neurointelligence, The University of Tokyo Institutes for Advanced Study, The University of Tokyo, Tokyo 113-0033, Japan; Science Groove Inc., Fukuoka 810-0041, Japan; Department of Medicine and Bioregulatory Science, Graduate School of Medical Sciences, Kyushu University, Fukuoka 812-8582, Japan

## Abstract

Acute liver failure (ALF) is a serious disease that progresses from acute liver injury (ALI) and that often leads to multiorgan failure and ultimately death. Currently, effective treatment strategies for ALF, aside from transplantation, remain elusive, partly because ALI is highly heterogeneous. Furthermore, clinicians lack a quantitative indicator that they can use to predict which patients hospitalized with ALI will progress to ALF and the need for liver transplantation. In our study, we retrospectively analyzed data from 319 patients admitted to the hospital with ALI. By applying a machine-learning approach and by using the SHapley Additive exPlanations (SHAP) algorithm to analyze time-course blood test data, we identified prothrombin time activity percentage (PT%) as a biomarker reflecting individual ALI status. Unlike previous studies predicting the need for liver transplantation in patients with ALF, our study focused on PT% dynamics. Use of this variable allowed us to stratify the patients with highly heterogeneous ALI into six groups with distinct clinical courses and prognoses, i.e. self-limited, intensive care–responsive, or intensive care–refractory patterns. Notably, these groups were well predicted by clinical data collected at the time of admission. Additionally, utilizing mathematical modeling and machine learning, we assessed the predictability of individual PT% dynamics during the early phase of ALI. Our findings may allow for optimizing medical resource allocation and early introduction of tailored individualized treatment, which may result in improving ALF prognosis.

**Significance Statement**

Acute liver failure (ALF) is a diverse syndrome with poor prognosis, and the lack of proper classification hinders understanding its pathophysiology and developing treatments. While liver transplantation may be necessary, organ scarcity and age restrictions are major challenges. Selecting patients and timing surgery are difficult due to variations in acute liver injury (ALI) progression to ALF. A promising strategy is predicting which patients with ALI will progress to ALF and initiating early interventions. This study aims to predict ALI progression at both group and individual levels during early ALI. Our findings have significant clinical implications for establishing early treatment plans, crucial for improving patient outcomes, treatment efficacy, and optimizing healthcare delivery.

## Introduction

Acute liver failure (ALF) is a serious disease characterized by coagulation disorders and encephalopathy that progresses from acute liver injury (ALI) and that often leads to multiorgan failure, culminating in death (1, 2). However, the heterogeneity of ALI and the significant individual variation in the progression to ALF make predicting clinical outcomes challenging. Additionally, ALF has few established treatments because its pathophysiology is not well understood (3, 4). Diagnostically, ALF has been characterized since the 1950s by a prolonged prothrombin time (PT; i.e. an international normalized ratio [INR] > 1.5) and a certain degree of mental status alteration (e.g. hepatic encephalopathy or hepatic coma) (5). In Japan, ALF is diagnosed by criteria established by the Intractable Hepato-Biliary Diseases Study Group and is classified as ALF with or without hepatic coma. ALF with hepatic coma is further subclassified into two disease types: acute and subacute according to the period during which hepatic coma develops (6).

Liver transplantation is nearly the sole recourse for patients with ALF, yet the global availability of liver transplants falls short of meeting the needs of all (7). Therefore, it is crucial to promptly identify patients who genuinely require transplantation so that medical resources can be optimally allocated among patients with ALF. So far, several models have been developed to predict the necessity of liver transplantation in patients with ALF at specific time points, such as at admission (8–10). Despite efforts primarily focusing on population averages and the prediction of severity of patients with ALF, few studies have analyzed a frame work for stratifying patients with heterogenous ALI to predict progression to ALF.

In this study, we quantitatively analyzed extensive clinical data from the first week of hospitalization in a cohort of 319 patients with ALI at Kyushu University Hospital retrospectively. Because a machine-learning approach indicated that PT activity percentage (PT%) mostly reflected individual ALI status, we focused on time-course patterns of PT% for the 7 days postadmission. Using this framework, we were able to stratify the patients with highly heterogeneous ALI into six groups with distinct clinical courses and prognoses, i.e. self-limited, intensive care–responsive, or intensive care–refractory patterns. Notably, we could predict these stratified groups upon admission and distinguish between patients who would require intensive care at a high-volume center and those who would require liver transplantation. Furthermore, using mathematical modeling and machine learning, we demonstrated that individual prediction of PT% dynamics for the 7 days postadmission is feasible upon admission. A better understanding of individual heterogeneity in ALI not only will contribute to tailored treatments that consider individual PT% dynamics, thus optimizing medical resource allocation, but will also provide clinical insights into understanding the mechanisms leading to severe ALF. Therefore, distinct from previous predictive models (8–11), our approach significantly enhances understanding of the pathophysiology of ALI/ALF and may improve treatment outcomes.

## Results

### Description of cohort and study design

We retrospectively analyzed extensive clinical data collected from 319 patients with ALI, who were admitted to Kyushu University Hospital between January 2007 and March 2021. The most frequent etiology was unknown (28.7%) followed by hepatitis B virus (25.6%), autoimmune hepatitis (14.4%), hepatitis A virus (11.9%), and drug-induced liver injury (4.7%). The mean age was 46.5 years and 52.2% were male. Among the 319 patients with ALI, 116 patients (8.2 patients per year on average) were diagnosed with ALF. Approximately 200 to 300 patients with ALF are seen in Japan annually (12, 13), and thus Kyushu University Hospital is considered to be one of the high-volume centers managing ALF in Japan. The clinical data, which were annotated so that we could assess individual clinical outcomes, included basic demographic information (such as age and sex), blood test results, treatment details, and other information (see Fig. S1, Tables S1 and S2 for details). We defined “clinical outcome” as whether patients hospitalized with ALI would survive without liver transplantation or not. Transplant-free survival (TFS) cases (*n* = 264) were categorized as those recovering without the need for liver transplantation, whereas non-TFS cases (*n* = 55) were those who died or required liver transplantation for survival. Further details on data preparation are outlined in the Methods.

### Exploring biomarkers for ALF

**Fig. 1.**
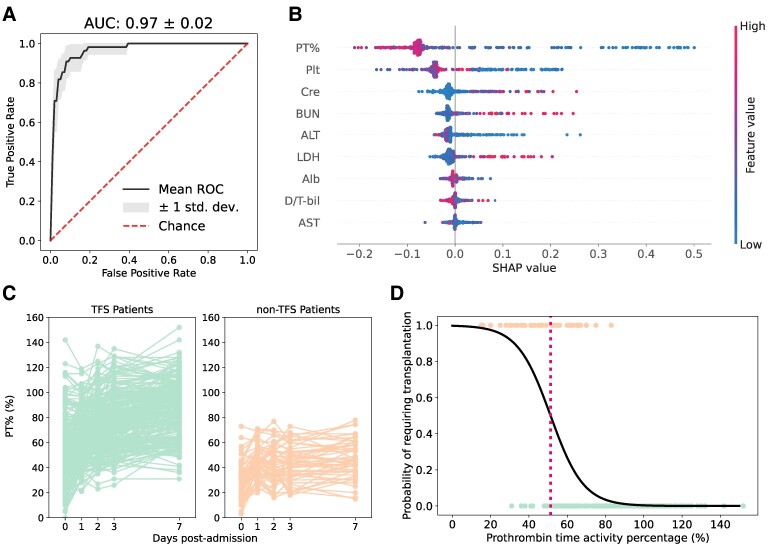
Exploring a biomarker for individual ALF progression: A) The ROC curve of RF classifiers trained to predict the need for transplantation based on the blood test data at 7 days postadmission is presented (using data from Fig. S1A). The corresponding ROC–AUC is calculated and displayed at the top of the panel. B) Feature importance of the predictive model in A is illustrated as a SHAP summary plot (using data from Fig. S1A). The *y*-axis represents the blood test items, arranged in order of their contribution to the prediction. The contribution of each feature for each patient (each point) to the prediction is represented as SHAP values (*x*-axis). A higher SHAP value means a higher contribution to the likelihood of the need for transplantation, while a lower value indicates a higher contribution to the likelihood of no need. The color of each point in each feature represents the value of that feature for the patient, with higher values shown in red and lower values in blue. PT%, prothrombin time activity percentage; Plt, platelet; Cre, creatinine; BUN, blood urea nitrogen; ALT, alanine aminotransferase; LDH, lactate dehydrogenase; Alb, albumin; D/T-bil, ratio of direct bilirubin (D-bil) to total bilirubin (T-bil); AST, aspartate aminotransferase. C) The time course of observed PT% values for all cases is depicted. The green and orange plots (i.e. left and right panels) represent the TFS (*n* = 264) and non-TFS (*n* = 55) patients, respectively (using data from Fig. S1B). D) A logistic regression model for predicting severe patients using the PT% values on day 7 is computed (using data from Fig. S1A). The green and orange dots (i.e., the dots with y-axis values of 0 and 1, respectively) represent the TFS and non-TFS patients, respectively. The red dashed line indicates the PT% threshold value (i.e. PT% = 51.30%).

To identify a biomarker for predicting non-TFS in patients hospitalized with ALI, we used the time-course blood test data collected on days 0, 1, 2, 3, and 7 after admission. We applied a machine-learning approach, specifically the random forest (RF) classifier (see Methods for details), to predict whether a given patient belonged to the TFS or non-TFS group based on blood test data measured on different dates (Fig. S1B). It is reasonable that the receiver operating characteristic (ROC)–area under the curves (AUCs) of RF classifiers show improvement over time after admission, as shown in Fig. S2A. In fact, our RF classifiers with the data at 7 days postadmission achieved an exceptionally high accuracy rate, with an ROC–AUC of 0.97 (Fig. 1A: sensitivity analyses on data preparation are shown in Fig. S2B). These results indicated that the blood test data may contain biomarker(s) associated with the clinical outcome we were interested in.

We then computed SHapley Additive exPlanations (SHAP) values (14–17), an index that quantifies the contribution of each item in the prediction, to evaluate the significance of nine blood markers collected at 7 days postadmission (i.e. data from Fig. S1A) in discerning between TFS and non-TFS patients. This computation was based on the highest ROC–AUC values. PT% (defined further below) emerged as the most crucial factor, with platelets, creatinine, blood urea nitrogen, alanine aminotransferase (ALT), lactate dehydrogenase (LDH), albumin, the ratio of direct to total bilirubin, and aspartate aminotransferase (AST) following in importance (Fig. 1B). In this study, we have quantified PT, a metric used in coagulation studies to assess the efficiency of the extrinsic coagulation pathway (a component of the clotting cascade (18)) as the PT activity percentage, hereafter referred to as PT%. At Kyushu University Hospital, the normal reference value of PT% is 70–130%. PT% is determined by contrasting the patient's PT result with a normal reference value (19), with lower PT% values indicating more severe conditions (see below). Note that PT results can be reported using other metrics such as PT, PT ratio, and INR (i.e. PT-INR) (20, 21). In Fig. S3, we used nonlinear regression to conduct a comparison between PT% and PT-INR, as well as between PT% and PT. This analysis revealed a strong correlation between these measurements, with mutual information coefficients of 0.96 and 0.89, respectively. Additionally, we observed that the PT% values for our patients with ALI covered a wider range (i.e. 0–150%) than those for PT-INR (i.e. 0–15%) or PT (i.e. 0–80%), suggesting that PT% is more robust to measurement error for mathematical modeling and data fitting (see below). Consequently, although INR is a standardized system for reporting PT that takes into account the sensitivity of the reagents used in the PT test (22), for the evaluation of PT in this study, we used PT% without a loss of generality.

The PT% value is acknowledged as a liver function measure strongly influenced by coagulation factors such as factors II, V, VII, and X, all of which are produced by the liver (23, 24). It was quite natural that the SHAP values revealed that patients with low PT% values had a higher likelihood of being non-TFS cases because PT% reflects the status of hepatic protein synthesis and is the defining parameter of ALF. Similarly, when we compared the time course of PT% values between the TFS and non-TFS patients (represented by green and orange curves, respectively; Fig. 1C), we observed two distinct patterns. Among the TFS patients, PT% values tended to increase to relatively high levels over time after admission, whereas PT% values remained low in the non-TFS patients.

Next, to establish a threshold for PT% values indicative of ALI status, we conducted a logistic regression analysis with PT% values at 7 days postadmission as the predictor variable and clinical outcome as the outcome variable (Fig. 1D). We achieved similarly high ROC–AUCs of 94% for non-TFS, even when excluding other blood markers. This suggested that the PT% value alone can serve as a biomarker of individual ALI status, at least at 7 days postadmission. In addition, we calculated cutoff values of PT% corresponding to a probability of 0.5 (51.30%; the red dashed line in Fig. 1D: this value corresponds to a PT-INR of 1.37). The first panel of Fig. S4 demonstrates that the threshold generally distinguishes between TFS and non-TFS patients at 7 days postadmission (*P* < 0.001 by Wilcoxon rank-sum test). Conversely, exceeding the threshold at any point before day 7 does not necessarily indicate non-TFS. Therefore, it is particularly important to differentiate non-TFS cases during the early phase of ALI by considering the dynamics of PT% after admission (see next section).

Regarding other biomarkers, we compared each value between the TFS and non-TFS patients as depicted in Fig. S4. We found that the non-TFS groups showed low PT% and albumin. These clinical findings indicate that treatment-unresponsive ALI may be linked to poor prognosis.

### Stratifying and characterizing ALF progression by PT% dynamics

**Fig. 2.**
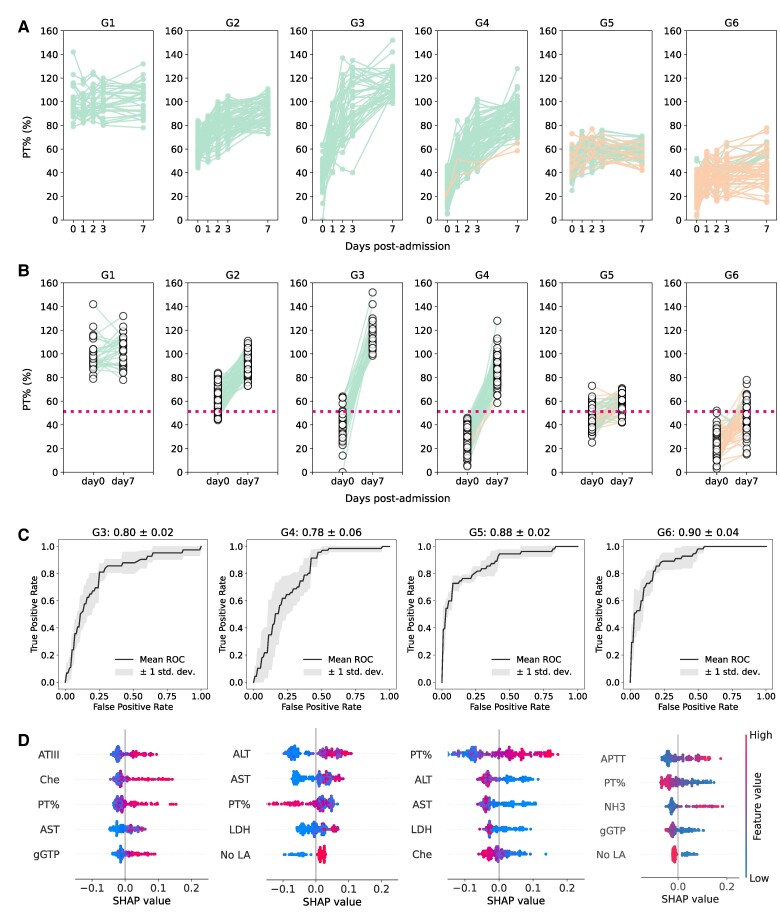
Stratifying and characterizing PT% dynamics during the progression of ALF: A) The time-course patterns of PT% for each group stratified by unsupervised time-series clustering are depicted and colored accordingly (using data from Fig. S1B). The green and orange plots represent the TFS (*n* = 264) and non-TFS (*n* = 55) patients. B) The PT% at admission and 7 days postadmission are compared across the stratified groups (using data from Fig. S1B) with colors corresponding to those used in A. The red dashed line indicates the PT% threshold value obtained in Fig. 1D (i.e. PT% = 51.30%). C) Shown is the ROC curve of the binary classification prediction (e.g. either G6 or not) using RF based on the clinical dataset on admission (using data from Fig. S1C) except for G1 and G2. The corresponding ROC–AUCs are calculated and displayed at the top of each panel. D) Feature importance of the predictive model in C is illustrated as a SHAP summary plot (using data from Fig. S1C). Here, only the top 5 features are shown, but the SHAP values for all features are presented in Fig. S4. ATIII, antithrombin III; Che, cholinesterase; PT%, prothrombin time activity percentage; AST, aspartate aminotransferase; gGTP, γ-glutamyl transpeptidase; ALT, alanine aminotransferase; LDH, lactate dehydrogenase; APTT, activated partial thromboplastin time; NH3, serum ammonia; no LA, no liver atrophy.

Considering the notable individual-level variation in the time-course patterns of PT% values over time at and after admission, as depicted in Fig. 1C, even among the TFS or non-TFS group, we further examined individual heterogeneity in PT% dynamics. We performed an unsupervised clustering analysis using dynamic time warping (25) to stratify the time-course patterns of PT% values into six groups (i.e. G1 = 31, G2 = 66, G3 = 42, G4 = 70, G5 = 55, and G6 = 55 patients, respectively; see Methods for details). Figure 2A illustrates the PT% dynamics of all the six groups, clearly demonstrating distinct time-course patterns, with most non-TFS patients stratified into G5 and G6 (i.e. 23.6 and 72.7% of patients in G5 and G6 were non-TFS, respectively). Note that the non-TFS cases (*n* = 2) in G4 showed PT% improvement in response to treatment and may have recovered without liver transplantation; however, they were transplanted early because a donor liver was available and their hepatic encephalopathy was persistent at that time. Interestingly, when comparing the PT% value threshold calculated in Fig. 1D, a consistent pattern emerged. The time-course PT% values of all patients categorized into G1 to G4 remained above the threshold at 7 days postadmission, even in cases where the PT% values were below the threshold at the time of admission (Fig. 2B). Significantly, individuals in G1 and G2 consistently maintained PT% values above the threshold over time without intensive care, such as plasma exchange, anticoagulation, or continuous hemodiafiltration (Fig. 2B and Table S2). In contrast, some patients in G5 and G6, 18.2 and 63.6%, respectively, had PT% values below the threshold at 7 days postadmission despite intensive care (Fig. 2B and Table S2). Although most patients in G3 and G4 presented PT% values below the threshold at the time of admission, intensive care drastically improved PT% values, which were above the threshold at 7 days postadmission (Fig. 2B and Table S2). Our stratification based on PT% dynamics successfully captured the need and the effect of intensive care at the group level, despite the large variation in PT% values at admission. Our results emphasize that the time-course patterns of PT%, rather than the PT% values at admission (except for patients in G1 and G2), are crucial for predicting and understanding the progression of ALF.

We then investigated whether each group could be predicted by the clinical datasets measured at admission, including the results of blood tests, basic information, and diagnostic details, as described in Fig. S1C. Subsequently, we focused our attention on G3 to G6 because PT% values at admission clearly distinguished whether a given patient belonged to G1/G2 or non-G1/G2. Similarly, we used RF classifiers and evaluated the ROC curve for binary classification prediction (e.g. either G6 or not). In general, we achieved high accuracies of ROC–AUC for predicting G3 (80%), G4 (78%), G5 (88%), and G6 (90%), as shown in Fig. 2C. As expected from the lower PT% values at admission for G3, G4, G5, and G6, the PT% value is one of the most crucial factors for prediction among the SHAP values (see Fig. 2D for the top 5 features and Fig. S5 for all features). Apart from the requirement for various factors like antithrombin III, cholinesterase, AST, and others to predict an individual's progression group, our RF classifiers suggest that, upon admission, it may be possible to predict the clinical outcome of patients hospitalized with ALI by determining which of the stratified groups a patient belongs to.

Regarding the blood test data selected as important features for G3 to G6 in Fig. 2D, we compared each value among the six groups as depicted in Fig. S6. We found that G3 and G4 showed higher AST, ALT, and LDH than G5 despite having similar PT%. G5 and G6 were characterized by liver atrophy and marked decrease of PT%, which may reflect severe liver dysfunction on admission.

### Predicting individual future PT% dynamics at admission

**Fig. 3.**
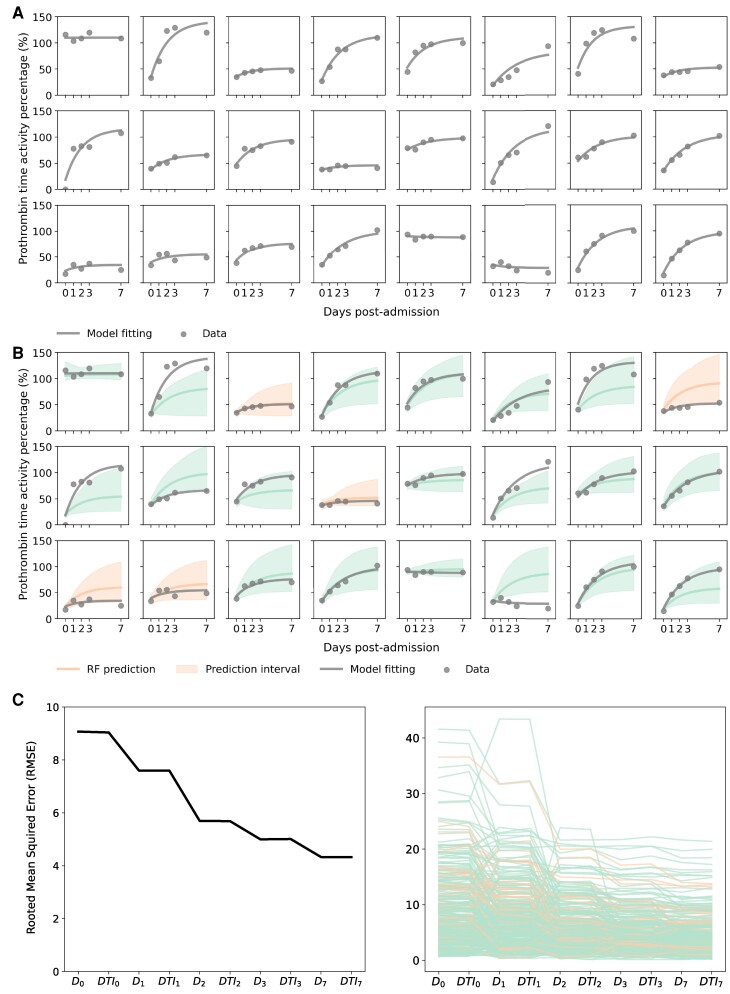
Predicting individual PT% dynamics during the early progression phases: A) The reconstructed individual PT% dynamics for 24 representative patients are displayed. The dots and black solid curves represent the observed PT% data and the best-fitted model by NLMEM based on the entire PT% dataset (i.e. model fitting), respectively (using data from Fig. S1B). B) The predicted PT% dynamics by the mathematical model with RF-predicted parameters based on the blood test data on admission for the 24 patients (i.e. RF prediction) are presented. The green and orange solid lines correspond to TFS and non-TFS cases, respectively, while the shaded area in each panel indicates the mean and 95% prediction interval of the model prediction, respectively (using data from Fig. S1C). The dots and black solid curves are the same as in A. C) The average and individual RMSEs between the observed PT% data and the RF prediction with different datasets are depicted in the left and right panels, respectively (using data from Fig. S1E). Note that $D_{0}$ represents clinical data on admission, including blood test data; $\mathrm{DT}I_{0}$ includes $D_{0}$ and treatment information on admission; $D_{t}$ includes $D_{0}$ and blood test data until day *t* postadmission; $\mathrm{DT}I_{t}$ includes $D_{t}$ and treatment information until day *t* postadmission. The green and orange plots represent TFS and non-TFS patients, respectively.

While our RF classifiers may determine the clinical outcome of patients with ALI at admission in terms of the stratified groups (i.e. G1 to G6) in Fig. 2C, there is a greater practical demand in clinical settings for making individual predictions about real-time future PT% dynamics (1, 22). To gain a deeper understanding of the individual heterogeneity among patients with ALI, we used a mathematical model describing the dynamics of PT% (Eq. 1 in Methods). We fitted the model to the time course of PT% values (illustrated in Fig. S1B), taking into account the inter-patient variability in parameters (see Methods for details, specifically labeled as “model fitting” in this study). We comprehensively reconstructed the PT% dynamics at the individual level for 7 days following admission, as illustrated for all individuals in Figs. 3A and S7, and summarized the estimated parameters in Table S3. We demonstrated that Eq. 1 well described the time course of PT% values over time after admission.

Next, we investigated the predictability of individual PT% dynamics during the early phase of ALI by combining mathematical modeling and machine learning. Specifically, we performed an RF regressor to estimate parameters in Eq. 1: we used the datasets at admission in Fig. S1C as predictor variables and the estimated parameter values obtained from model fitting in Table S3 as the outcome variables (see Methods for details, specifically labeled as “RF prediction”). The detailed comparison between the estimated and predicted parameters is explored in Fig. S8 (sensitivity analyses on data preparation are referred to in Fig. S2C). We depicted the predicted PT% dynamics using the mathematical model with “RF-predicted parameters” in Figs. 3B and S7A, and obtained a determination coefficient of 0.85 between the model fitting and RF prediction at day 0, 1, 2, 3, and 7 postadmission. These findings suggest that individual prediction of PT% dynamics for the 7 days postadmission is viable using the clinical datasets obtained at admission (see below for further analysis).

At last, we examined whether enhancing the RF predictions in Fig. 3B was achievable by including clinical data, encompassing treatment information alongside the admission data. We expanded the admission data to include day-by-day blood test results and treatment details up to day 7 postadmission (as outlined in Fig. S1E) and performed the RF regressor, as previously described. The left and right panels in Fig. 3C showed the average and individual root mean squared errors (i.e. RMSEs) between the observed PT% data and the RF prediction with different datasets, respectively. Interestingly, as depicted in Figs. S5B and S9, the predicted PT% dynamics, based on RF prediction using blood test data until 2 days postadmission, showed significant improvement, with narrower 95% prediction intervals. While the RMSEs showed significant improvement up to 2 days postadmission, the inclusion of treatment details did not impact the predictions. In fact, by day 2 postadmission, there was a reduction of 68.9% in the total RMSE difference from admission to 7 days postadmission. These findings suggest that the clinical outcome of patients hospitalized with ALI is primarily determined up to 2 days postadmission when all patients are treated uniformly. We address this further in the Discussion.

Furthermore, we similarly assessed the RF predictions within the stratified groups (i.e. G1 to G6) as shown in Fig. S10. While we observed similar trends across all the groups, the RMSEs in G3 were larger than those in other groups. These results also indicated that predicting the intermediate patterns of PT% dynamics at the individual level becomes more challenging even with the addition of clinical datasets.

## Discussion

Using longitudinal clinical data collected from 319 patients with ALI who had been admitted to Kyushu University Hospital, we analyzed clinical variables to assess whether the patients would survive without liver transplantation. Unlike previous observational studies of ALI or ALF which have attempted to predict the need for liver transplantation by using clinical variables, including ALF etiologies upon admission (8, 9, 26), we focused on time-course patterns of PT% values. In this way, we were able to stratify the patients with highly heterogeneous ALI into six distinct clinical groups: G1 to G6. All non-TFS patients, except for the two non-TFS cases stratified in G4 who underwent early transplantation because of the availability of a donor liver, were stratified into G5 and G6. Whereas all patients in G1 to G4 exhibited PT% values higher than the threshold of 51.30% at 7 days postadmission, 40.9% of the patients in G5 and G6 had lower PT% values. Our results indicate that these groups stratified by PT% dynamics sufficiently reflect the prognosis of patients with ALI.

As patients in G1 and G2 presented PT% values consistently above the threshold without intensive care such as plasma exchange or continuous hemodiafiltration, they could be followed up at general hospitals (self-limited patients). Most patients in G3 and G4 presented PT% values below the threshold at the time of admission; however, intensive care drastically improved PT% values, indicating that these patients should be treated at a high-volume center (intensive care–responsive patients). A substantial number of patients in G5 and G6 had PT% values below the threshold at 7 days postadmission despite intensive care, which implies that liver transplantation should be considered early in these patients (intensive care–refractory patients).

As a decline of PT% generally proceeds hepatic encephalopathy under deteriorating hepatic function, ALI first advances to ALF without coma and then to ALF with coma. According to the proportion of patients with ALI, ALF without coma, and ALF with coma in each group shown in Table S2, the patients in G2, G4, and G6 seemed to be more advanced than those in G1, G3, and G5, respectively. Our method to stratify patients with ALI by PT% dynamics is supposed to reflect the need and the effect of intensive care. In addition, our stratification indicates the pathophysiological characteristics of each group. Given that liver atrophy appears when ALF with coma continues, the high proportion of liver atrophy in G5 and G6 indicates that the poor prognosis of these groups might be attributable to their severely advanced status (Fig. S6 and Table S2). Furthermore, many groups reported that the hepatic microcirculatory disturbance caused by sinusoidal hypercoagulation is involved in ALF pathogenesis (27–31). We reported previously that patients with a high ALT/LDH ratio also have hepatic microcirculatory disturbance (32) and tend to respond to intensive care, including steroid pulse therapy and anticoagulant therapy (33). Meanwhile, certain patients did not show the elevation of coagulation markers, and these patients differed in the elevation of interferon-gamma (32–34). Similarly, the inflammatory cells and cytokines were known to be involved in ALF pathogenesis and correlate with ALF prognosis (35–38). These findings indicated the several subgroups of ALF with different underlying mechanisms. At present, there is no classification of ALF based on pathogenesis, and our stratification may help us to establish a new classification. In fact, the proportion of patients with an ALT/LDH ratio of <1.5 in G3 and G4 was higher than in the other groups (Table S2) and intensive care drastically increased PT% in these groups (Fig. 2B), which is consistent with our previous reports. Taken together, the stratification of patients with ALI/ALF in this study holds mechanistic significance, and further analysis of the differences between each group is expected to offer new insights into the mechanisms underlying non-TFS.

The heterogeneity of ALF etiology and regional differences may lead to different inspections among experts. This lack of objectivity and generalizability may hinder the development of basic and clinical research on ALF and the development of new treatments. It is important to emphasize that our cohort includes all stages of ALF progression, from self-limited ALI to ALF with coma. And we have successfully stratified these highly heterogeneous patients (using unsupervised machine learning) according to their disease progression dynamics based solely on PT% dynamics, and have predicted with high accuracy whether they will be self-limited (G1 and G2), intensive care responsive (G3 and G4), or intensive care refractory (G5 and G6) at the time of admission. This will enable nonspecialists to predict treatment responsiveness as well as specialists and make appropriate decisions about hospital transfers in the case of ALF, a relatively rare condition for which few hepatologists can accurately grasp the condition.

Some observational studies, such as the King's College Criteria (9), Acute Liver Failure Study Group Prognostic Index (ALFSG-PI) (8), and a decision tree model (10) have been designed to predict the need for liver transplantation using clinical data at the time of admission. In contrast, the ALF early dynamic model (26) for non-acetaminophen-induced patients with ALF (non-APAP-ALF) and the binary mixed model (BiMM) forest model (11) for patients with APAP-ALF utilized sequential clinical data postadmission to predict the need for liver transplantation. However, none of these studies focused on PT% dynamics because their objectives were patients with ALF with coma, whose PT% might severely decline on admission. In contrast, the present study analyzed patients with ALI and stratified them by PT% dynamics into six groups that sufficiently reflected prognosis and treatment responsiveness. Notably, these groups stratified by PT% dynamics could be predicted by using the clinical datasets measured at admission, including the results of blood tests, basic demographic information, and diagnostic details. Therefore, we could predict who might need or respond to intensive care at the time of admission. Our findings may allow for optimizing medical resource allocation and early introduction of tailored individualized treatment plans, which may result in improving ALI prognosis. In fact, only 3 patients among 88 patients whose PT% was above 40% in G3/G4/G5/G6 advanced to ALF whose PT% was below 40% in our cohort. Interestingly, through the combination of mathematical modeling and machine learning, we have demonstrated the potential for real-time individual prediction of PT% dynamics for the 7 days postadmission using clinical datasets obtained at admission. Additionally, we found that the addition of clinical datasets up to 2 days postadmission significantly improved the predictions. Although predicting the patterns of PT% dynamics, particularly for patients belonging to G3, is more challenging, our real-time prediction with admission data, including day-by-day blood test results up to 2 days postadmission, provides notable information, at least for the first 7 days. In clinical settings, if individual predictions regarding real-time future PT% dynamics are updated day-by-day, we will be able to select the patients truly requiring liver transplantation and thus reduce the number of unnecessary liver transplantations, which in turn would benefit the many other patients awaiting brain-dead donor livers.

The model proposed in this study differs significantly from previous models in that it provides stratification that focuses on the dynamics of disease progression and group prediction at the time of admission, rather than on whether the patient can survive without transplantation. Therefore, it is difficult to strictly compare it with previous models due to differences in the characteristics of the cases treated and the predicted outcomes (see Table S4 for details). However, the performance of the G5 and G6 predictions (ROC–AUC: 0.88 and 0.9, respectively; see Fig. 2C) or the prediction of survival without transplantation using only admission blood test data (ROC–AUC: 0.85; see Fig. S2A) can serve as points of comparison with earlier models.

Our study has several limitations. First, our analysis relied on data from 319 patients at a single high-volume center. In recent studies with extensive data from the ALFSG, models were developed to predict severity by multivariate analysis of admission data from >1,900 patients (8). However, those studies were limited to a dataset collected at a specific time point (at admission). In another study using the BiMM forest model (11), although a time-series blood dataset was used, the study focused on predicting daily encephalopathy grades and lacked a framework for the early prediction of ALF progression. While the number of patients in our study is limited compared with large cohort studies, our extensive longitudinal clinical data include time-course blood test data, including PT% values. Therefore, we achieved stratification of ALI by the need for and responsiveness to intensive care. Furthermore, we achieved real-time predictions of future PT% dynamics, thus addressing early prediction of ALI progression through an integrative approach that combines process-based mathematical modeling and machine learning. Secondly, further investigation is needed to explore the association between the accuracy of our real-time prediction and treatment details such as plasma exchanges, steroid pulse therapy, and anticoagulant therapy. Our analysis demonstrated that the inclusion of treatment details did not impact the predictions. This might be attributed to the uniform and high-quality treatment provided to all patients at a single high-volume center. This aspect is particularly relevant in real-world clinical settings, where treatments may differ among hospitals or countries according to the experiences of frontline clinicians. We are currently planning to collect prospective and multicenter data. This will allow us to quantitatively evaluate the impact of treatment on PT% and outcomes despite patient heterogeneity as the amount of data increases. Finally, in our study, it is possible that population characteristics influenced the analysis. Because Kyushu University is a transplant hospital, the number of severe ALI cases was comparatively high. Additionally, there are regional differences and changes over time in ALI etiologies (13). These variances may affect clinical outcome. However, because the population analyzed in this study included major causes of ALI, such as autoimmune hepatitis, hepatitis A and hepatitis B virus infections, and drug-induced liver injury, our analysis may have broad applicability for ALI. These challenges need to be considered when dealing with larger datasets, including validation data from different cohorts (e.g. multicenter cohort study) as they become available.

In conclusion, this study is the first to analyze PT% dynamics in patients with ALI and then derive a stratification model to identify six groups reflecting the need for and responsiveness to intensive care. Notably, these stratified groups could be predicted using the clinical dataset upon admission, which will enable optimizing medical resource allocation and early introduction of tailored individualized treatment plans, resulting in improving ALF prognosis. Furthermore, we plan to make our stratification model public for clinicians treating ALF in the future, and that will enable them to identify appropriate treatment for each subgroup during the early phase. Additionally, our clinical stratification at admission and real-time predictions of future PT% dynamics open a door for preemptive medicine against severe ALF and the ability to tailor treatment plans at the individual level.

## Methods

### Ethics statement

This study was performed in accordance with the Declaration of Helsinki and was approved by the Ethics Committees of Nagoya University (hc23-13) and Kyushu University Hospital (nos 222283-01 and 23202-00). Written informed consent was waived because of the retrospective design. Consent for publication was obtained from all patients.

### Study data

The present study was a single-center retrospective study in patients with ALI admitted at Kyushu University Hospital between January 2007 and March 2021. Clinical data were collected from medical records. ALF was diagnosed by using criteria established by the Intractable Hepato-Biliary Diseases Study Group in Japan (6). Patients with previously normal liver function who had a prothrombin activity percentage of 40% or less of the standardized value or an INR of 1.5 or more caused by severe liver damage within 8 weeks of the onset of symptoms were diagnosed as having ALF. Patients with ALF were further classified into ALF with or without hepatic coma. ALF with hepatic coma was further subclassified into two disease types, the acute type and the subacute type, which were defined as grade II or more severe hepatic encephalopathy developing within 10 days or between 11 and 56 days after the onset of disease symptoms, respectively. As the definitions of ALF in some countries require hepatic coma (12), ALF with coma in the Japanese definition is the same as ALF in these countries. ALF without coma in the Japanese definition is considered to be the earlier condition of ALF with coma and is a practical disease entity for analyzing the contiguous progression from ALI to ALF with coma. Patients showing PT% values of <40% of the standardized value or an INR of 1.5 or more and grade II or more severe hepatic coma between 8 and 24 weeks after the onset of disease symptoms were diagnosed as having late-onset hepatic failure. Patients with malignant tumors and liver cirrhosis were excluded.

Regarding the therapeutic strategy, the hepatology team at Kyushu University decided how to treat each patient. Plasma exchange for the treatment of ALF is recommended in the Clinical Practical Guideline published by the European Association for the Study of the Liver (EASL) (39) and has been considered a standard treatment for ALF globally (40). Plasma exchange was repeated to keep PT% over 40% or PT-INR <1.5, and hemodiafiltration was performed if necessary. In Japan, because of the lack of brain-dead donors, patients with ALF frequently undergo medical treatments other than liver transplantation, such as high-dose intravenous methylprednisolone therapy (steroid pulse therapy) and anticoagulation therapy (13). For high-dose intravenous methylprednisolone therapy (steroid pulse therapy), 1,000 mg of methylprednisolone was administered intravenously for 1 h/day for 3 days. Transcatheter arterial steroid injection therapy, which was reported as a new treatment for ALF (33, 41), was performed by injecting methylprednisolone via the proper hepatic artery over 1 h (1,000 mg/day for 3 days). Anticoagulant therapies, including recombinant thrombomodulin alfa, antithrombin III, and/or danaparoid sodium, were administered according to drug information. Liver transplantation was implemented regardless of the treatment method when liver insufficiency progressed and an appropriate donor was available.

### Data preparation

We used clinical data collected from 319 patients, incorporating 75 numerical variables as presented in Table S1 and 19 categorical variables as detailed in Table S2. Each variable had <20% missing values, as we excluded data with >20% missing values. Additionally, we converted categorical data into dummy variables. All the data, encompassing the 75 numerical and 19 categorical variables, underwent multiple imputation using R's mice package (42) to address missing values. This imputed dataset served as a representative set for our analysis. To assess the sensitivity of the imputation, we generated 50 different datasets and confirmed the robustness of our analysis, as shown in Fig. S1BC.

### Supervised machine learning for predicting clinical outcome

We used an RF classifier (43), a supervised machine-learning algorithm, to predict the clinical outcome of patients hospitalized with ALI. We approached the problem as a binary classification task, where the model aimed to predict whether a given patient's clinical status was considered severe, such as the necessity for liver transplantation, or not. We used the RandomForestClassifier from Python's scikit-learn and used the K-Fold cross-validation method (StratifiedKfold, Python, scikit-learn). Specifically, we split the data into five parts, used each for testing, and averaged the results to plot the ROC curve and to calculate the AUC. To calculate the SD, we used each fold's ROC values. Regarding the issue of data imbalance in the classifier, we addressed it using the synthetic minority over-sampling technique (SMOTE; Python, Imbalanced-learn).

### Logistic regression for predicting clinical outcome

Using logistic regression, we constructed a predictive model for individuals who develop severe conditions (i.e. non-TFS patients). In this model, we used PT% values on day 7 postadmission as the predictor variable while categorizing patients into TFS and non-TFS outcomes. We implemented this using Python's scikit-learn library and established a threshold for PT% values. The threshold was determined at the PT% value corresponding to a probability of 0.5 in the model that used all the data as training data. Regarding the calculation of ROC–AUC and the issue of data imbalance, we used the K-Fold cross-validation method and SMOTE as in the RF model, respectively.

### Unsupervised clustering and stratification of PT% dynamics

To stratify the time-course patterns of PT% values (i.e. PT% dynamics), we used dynamic time warping (25) to assess dissimilarity among PT% dynamics, utilizing Python's tslearn library. The number of clusters was determined through the elbow method, considering the sum of squared errors of prediction.

### Modeling PT% dynamics

To describe PT% dynamics, we used the following simple model:


(1) $$\frac{dP(t)}{dt}=g-DP(t),$$

where P(t) is the PT% value at time *t*, and parameters *g* and *D* represent an increase and decrease in PT% time, respectively. These changes are attributed to the supply and consumption (and degradation) of coagulation factors, including factors II, V, VII, and X (23, 24).

#### Quantifying PT% dynamics: model fitting

For the parameter estimation of Eq. 1 (i.e. *g*, *D*, and P(0)) and fitting of the time-course PT% values (referred to as “model fitting” in this study), we used a nonlinear mixed-effects modeling approach, which incorporates fixed effects as well as random effects describing the inter-patient variability in parameter values. Each parameter of patient *k*, $\theta_{k}(=\theta \times e^{\pi_{k}})$, is decomposed as the product of *θ* (the fixed effect) and $e^{\pi_{k}}$ (the random effect), where $\pi_{k}$ is assumed to be drawn from a normal distribution: N(0,Ω). The fixed effect parameters and random effect parameters were estimated by using the stochastic approximation expectation/maximization algorithm and empirical Bayes method, respectively. MONOLIX 2021R2 (www.lixoft.com) (44), a program for maximum likelihood estimation for nonlinear mixed-effects models, was used to fit the model to the time-course PT% values (Fig. 3A). The estimated parameters are summarized in Table S3.

#### Predicting PT% dynamics: RF prediction

Alternatively, for parameter estimation in Eq. 1 and the prediction of time-course PT% values (referred to as “RF prediction” in this study), we used an RF regressor, specifically the RandomForestRegressor from Python's scikit-learn library. In this model, the datasets at admission in Fig. S1C were used as predictor variables, with the estimated parameter values obtained from model fitting in Table S3 serving as the outcome variables. The individual parameter values were then estimated and referred to as “RF-predicted parameters” in this study. Note that we used the LeaveOneOut module in Python's scikit-learn library, training on all cases except one. The predicted PT% dynamics, along with its 95% prediction interval, were calculated using Eq. 1 with the RF-predicted parameters (see Fig. 3B). The 95% prediction interval was derived from the predicted values of each decision tree within the RF.

### Statistical analysis

When necessary, variables were compared among different groups using Fisher's exact test with residual analysis for categorical variables and ANOVA (for more than two groups) with the Bonferroni-corrected Wilcoxon rank-sum test or Wilcoxon rank-sum test (for two groups) for numerical variables. All statistical analyses were performed using R (version 4.2.0).

## Supplementary Material

pgaf004_Supplementary_Data

## Data Availability

The clinical data of the patients with ALF/ALI used in this study and the code we used for analyses are available via following GitHub URL: https://github.com/rik-yosh/R-Yoshimura_ALI/tree/master.

## References

[1] Bernal W , Wendon J. 2013. Acute liver failure. N Engl J Med. 369:2525–2534. 24369077 10.1056/NEJMra1208937

[2] Fujiwara K , et al 2008. Fulminant hepatitis and late onset hepatic failure in Japan. Hepatol Res. 38:646–657. 18328067 10.1111/j.1872-034X.2008.00322.x

[3] Polson J , Lee WM; American Association for the Study of Liver Disease. 2005. AASLD position paper: the management of acute liver failure. Hepatology. 41:1179–1197. 15841455 10.1002/hep.20703

[4] Stravitz RT , et al 2023. Future directions in acute liver failure. Hepatology. 78:1266–1289. 37183883 10.1097/HEP.0000000000000458 PMC10521792

[5] Trey C , Davidson CS. 1970. The management of fulminant hepatic failure. Prog Liver Dis. 3:282–298. 4908702

[6] Mochida S , et al 2011. Diagnostic criteria of acute liver failure: a report by the Intractable Hepato-Biliary Diseases Study Group of Japan. Hepatol Res. 41:805–812. 21884340 10.1111/j.1872-034X.2011.00860.x

[7] Goldaracena N , Cullen JM, Kim DS, Ekser B, Halazun KJ. 2020. Expanding the donor pool for liver transplantation with marginal donors. Int J Surg. 82S:30–35. 32422385 10.1016/j.ijsu.2020.05.024

[8] Koch DG , Tillman H, Durkalski V, Lee WM, Reuben A. 2016. Development of a model to predict transplant-free survival of patients with acute liver failure. Clin Gastroenterol Hepatol. 14:1199–1206.e2. 27085756 10.1016/j.cgh.2016.03.046 PMC6055510

[9] O'Grady JG , Alexander GJ, Hayllar KM, Williams R. 1989. Early indicators of prognosis in fulminant hepatic failure. Gastroenterology. 97:439–445. 2490426 10.1016/0016-5085(89)90081-4

[10] Speiser JL , Lee WM, Karvellas CJ; US Acute Liver Failure Study Group. 2015. Predicting outcome on admission and post-admission for acetaminophen-induced acute liver failure using classification and regression tree models. PLoS One. 10:e0122929. 25885260 10.1371/journal.pone.0122929 PMC4401567

[11] Speiser JL , et al 2019. Predicting daily outcomes in acetaminophen-induced acute liver failure patients with machine learning techniques. Comput Methods Programs Biomed. 175:111–120. 31104700 10.1016/j.cmpb.2019.04.012 PMC6530588

[12] Sugawara K , Nakayama N, Mochida S. 2012. Acute liver failure in Japan: definition, classification, and prediction of the outcome. J Gastroenterol. 47:849–861. 22825549 10.1007/s00535-012-0624-x PMC3423565

[13] Nakao M , et al 2018. Nationwide survey for acute liver failure and late-onset hepatic failure in Japan. J Gastroenterol. 53:752–769. 29030713 10.1007/s00535-017-1394-2

[14] Wang R , et al 2024. Deep learning-based identification of eyes at risk for glaucoma surgery. Sci Rep. 14:599. 38182701 10.1038/s41598-023-50597-0 PMC10770345

[15] Huang W , Zhu JY, Song CY, Lu YQ. 2024. Machine learning models for early prediction of potassium lowering effectiveness and adverse events in patients with hyperkalemia. Sci Rep. 14:737. 38184719 10.1038/s41598-024-51468-y PMC10771443

[16] Kawakami E , et al 2023. Monitoring of blood biochemical markers for periprosthetic joint infection using ensemble machine learning and UMAP embedding. Arch Orthop Trauma Surg. 143:6057–6067. 37115242 10.1007/s00402-023-04898-8

[17] Price GD , Heinz MV, Song SH, Nemesure MD, Jacobson NC. 2023. Using digital phenotyping to capture depression symptom variability: detecting naturalistic variability in depression symptoms across one year using passively collected wearable movement and sleep data. Transl Psychiatry. 13:381. 38071317 10.1038/s41398-023-02669-y PMC10710399

[18] Triplett DA . 2000. Coagulation and bleeding disorders: review and update. Clin Chem. 46:1260–1269. 10926920

[19] 1985. International Committee for Standardization in Haematology, International Committee on Thrombosis and Haemostasis: ICSH/ICTH recommendations for reporting prothrombin time in oral anticoagulant control. Thromb Haemost. 53:155–156. 3992515

[20] Glavinic R , et al 2022. Acute arterial thrombosis of lower extremities in COVID-19 patients. J Clin Med. 11:1538. 35329864 10.3390/jcm11061538 PMC8949095

[21] Tripodi A , Lippi G, Plebani M. 2016. How to report results of prothrombin and activated partial thromboplastin times. Clin Chem Lab Med. 54:215–222. 26351955 10.1515/cclm-2015-0657

[22] Robert A , Chazouilleres O. 1996. Prothrombin time in liver failure: time, ratio, activity percentage, or international normalized ratio? Hepatology. 24:1392–1394. 8938167 10.1053/jhep.1996.v24.pm0008938167

[23] Levy JH , Szlam F, Wolberg AS, Winkler A. 2014. Clinical use of the activated partial thromboplastin time and prothrombin time for screening: a review of the literature and current guidelines for testing. Clin Lab Med. 34:453–477. 25168937 10.1016/j.cll.2014.06.005

[24] Rossaint R , et al 2023. The European guideline on management of major bleeding and coagulopathy following trauma: sixth edition. Crit Care. 27:80. 36859355 10.1186/s13054-023-04327-7 PMC9977110

[25] Sakoe H , Chiba S. 1978. Dynamic programming algorithm optimization for spoken word recognition. IEEE Trans Acoust Speech Signal Process. 26:43–49.

[26] Kumar R , et al 2012. Prospective derivation and validation of early dynamic model for predicting outcome in patients with acute liver failure. Gut. 61:1068–1075. 22337947 10.1136/gutjnl-2011-301762

[27] Kato J , et al 2013. Interferon-gamma-mediated tissue factor expression contributes to T-cell-mediated hepatitis through induction of hypercoagulation in mice. Hepatology. 57:362–372. 22936459 10.1002/hep.26027

[28] Miyazawa Y , Tsutsui H, Mizuhara H, Fujiwara H, Kaneda K. 1998. Involvement of intrasinusoidal hemostasis in the development of concanavalin A-induced hepatic injury in mice. Hepatology. 27:497–506. 9462649 10.1002/hep.510270225

[29] Hirata K , Ogata I, Ohta Y, Fujiwara K. 1989. Hepatic sinusoidal cell destruction in the development of intravascular coagulation in acute liver failure of rats. J Pathol. 158:157–165. 2754546 10.1002/path.1711580211

[30] Fujiwara K , et al 1988. Intravascular coagulation in acute liver failure in rats and its treatment with antithrombin III. Gut. 29:1103–1108. 3410335 10.1136/gut.29.8.1103 PMC1433908

[31] Rake MO , Flute PT, Pannell G, Williams R. 1970. Intravascular coagulation in acute hepatic necrosis. Lancet. 1:533–537. 4190350 10.1016/s0140-6736(70)90767-1

[32] Kuwano A , et al 2021. Microcirculatory disturbance in acute liver injury. Exp Ther Med. 21:596. 33884034 10.3892/etm.2021.10028 PMC8056117

[33] Kuwano A , et al 2023. Transcatheter arterial steroid injection therapy improves the prognosis of patients with acute liver failure. Medicine (Baltimore). 102:e33090. 36897684 10.1097/MD.0000000000033090 PMC9997803

[34] Kurokawa M , et al 2024. Microcirculatory disturbance in acute liver injury is triggered by IFNgamma-CD40 axis. J Inflamm (Lond). 21:23. 38907339 10.1186/s12950-024-00387-w PMC11191181

[35] dos Santos DC , et al 2012. Activated lymphocytes and high liver expression of IFN-gamma are associated with fulminant hepatic failure in patients. Liver Int. 32:147–157. 22098464 10.1111/j.1478-3231.2011.02654.x

[36] Nguyen NT , Umbaugh DS, Sanchez-Guerrero G, Ramachandran A, Jaeschke H. 2022. Kupffer cells regulate liver recovery through induction of chemokine receptor CXCR2 on hepatocytes after acetaminophen overdose in mice. Arch Toxicol. 96:305–320. 34724096 10.1007/s00204-021-03183-0 PMC8762790

[37] Umbaugh DS , et al 2024. The chemokine CXCL14 is a novel early prognostic biomarker for poor outcome in acetaminophen-induced acute liver failure. Hepatology. 79:1352–1364. 37910653 10.1097/HEP.0000000000000665 PMC11061265

[38] Vazquez JH , et al 2022. Proteomics indicates lactate dehydrogenase is prognostic in acetaminophen-induced acute liver failure patients and reveals altered signaling pathways. Toxicol Sci. 187:25–34. 35172013 10.1093/toxsci/kfac015 PMC9216044

[39] European Association for the Study of the Liver . Electronic address: easloffice@easloffice.eu, et al 2017. EASL Clinical Practical Guidelines on the management of acute (fulminant) liver failure. J Hepatol. 66:1047–1081. 28417882 10.1016/j.jhep.2016.12.003

[40] Fernandez J , Bassegoda O, Toapanta D, Bernal W. 2024. Acute liver failure: a practical update. JHEP Rep. 6:101131. 39170946 10.1016/j.jhepr.2024.101131 PMC11337735

[41] Kotoh K , et al 2006. Arterial steroid injection therapy can inhibit the progression of severe acute hepatic failure toward fulminant liver failure. World J Gastroenterol. 12:6678–6682. 17075983 10.3748/wjg.v12.i41.6678 PMC4125675

[42] van Buuren S , Groothuis-Oudshoorn K. 2011. Mice: multivariate imputation by chained equations in R. J Stat Softw. 45:1–67.

[43] Breiman L . 2001. Random forests. Mach Learn. 45:5–32.

[44] Traynard P , Ayral G, Twarogowska M, Chauvin J. 2020. Efficient pharmacokinetic modeling workflow with the monolixsuite: a case study of remifentanil. CPT Pharmacometrics Syst Pharmacol. 9:198–210. 32036625 10.1002/psp4.12500 PMC7180005

