# Supplementary material for: Stratifying and predicting progression to acute liver failure during the early phase of acute liver injury
Source: PNAS Nexus. 2025 Feb 6;4(2):pgaf004. doi: 10.1093/pnasnexus/pgaf004 (PMC11801268; doi:10.1093/pnasnexus/pgaf004)
Supplement: pgaf004_Supplementary_Data [file pgaf004_supplementary_data.pdf]

Supplementary Information

Stratifying and predicting progression to acute liver failure during the early phase of acute liver injury

Raiki Yoshimura[1,†], Masatake Tanaka[2,†], Miho Kurokawa[2,†], Naotoshi Nakamura[1,‡], Takeshi Goya[2,‡], Koji Imoto[2], Motoyuki Kohjima[3,‡], Katsuhito Fujiu[4], Shingo Iwami[1,5,6,7,8,9,10,#,*] and Yoshihiro Ogawa[2,#,*]

[1]interdisciplinary Biology Laboratory (iBLab), Division of Natural Science, Graduate School of Science, Nagoya University, Nagoya, Japan. [2]Department of Medicine and Bioregulatory Science, Graduate School of Medical Sciences, Kyushu University, Fukuoka, Japan. [3]Department of Gastroenterology, NHO Kyushu Medical Center, Fukuoka, Japan. [4]Department of Cardiovascular Medicine, Graduate School of Medicine, The University of Tokyo. [5]Institute of Mathematics for Industry, Kyushu University, Fukuoka, Japan. [6]Institute for the Advanced Study of Human Biology (ASHBi), Kyoto University, Kyoto, Japan. [7]Interdisciplinary Theoretical and Mathematical Sciences Program (iTHEMS), RIKEN, Saitama, Japan. [8]NEXT-Ganken Program, Japanese Foundation for Cancer Research (JFCR), Tokyo, Japan. [9]International Research Center for Neurointelligence, The University of Tokyo Institutes for Advanced Study, The University of Tokyo, Tokyo, Japan. [10]Science Groove Inc., Fukuoka, Japan.

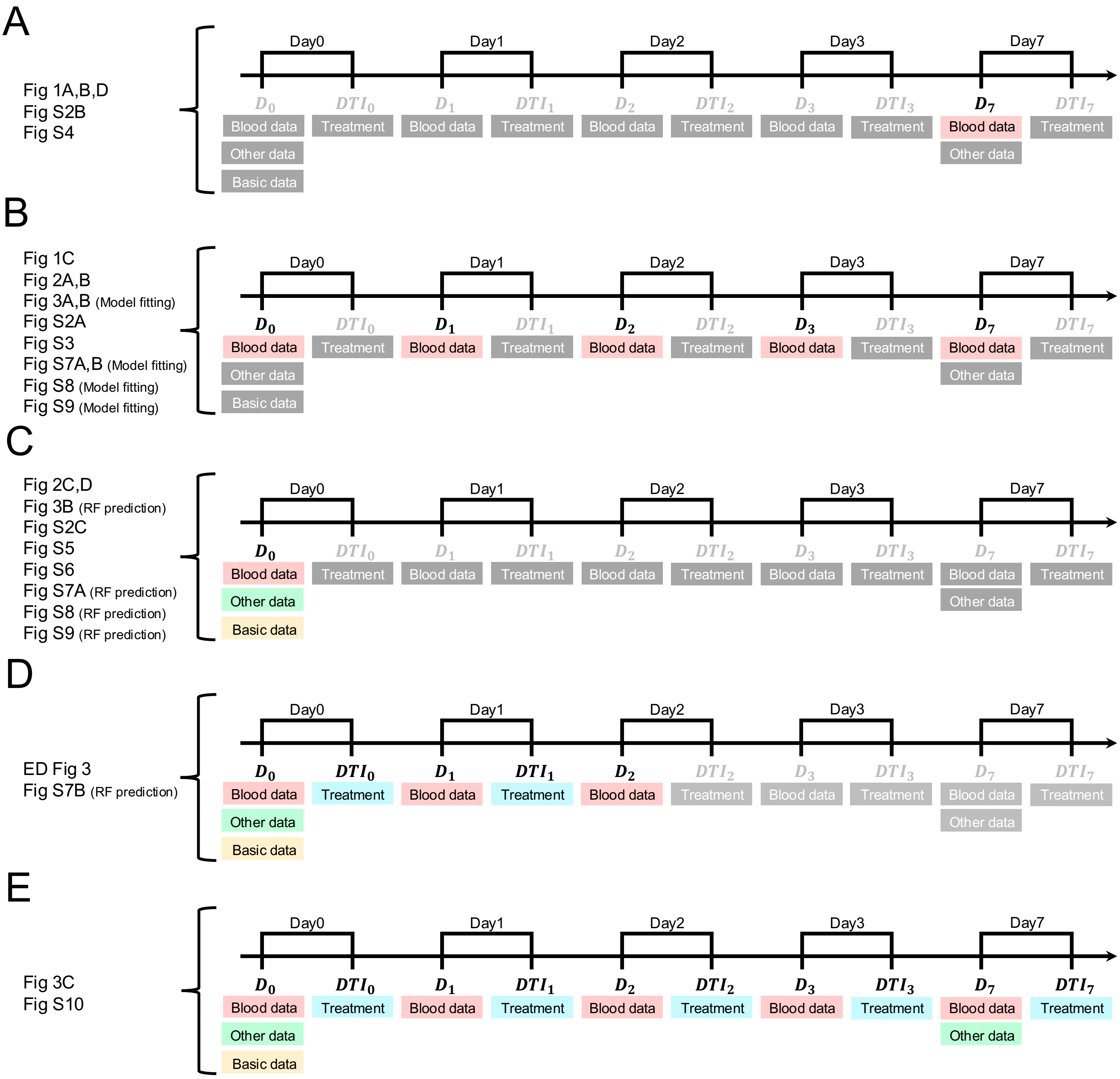

Supplementary Figure 1 | List of datasets utilized in our analysis: Three types of data (i.e., blood data, basic data, and other data) were used. The breakdown of each type of data is shown in **Table S1** and **S2**. **(A)**, **(B)**, **(C)**, **(D)**, and **(E)** represent the blood data from 7 days post-admission, the entire blood data (i.e., at day 0, 1, 2, 3, and 7 after admission), admission test data, test data until 2 days post-admission, and all time-series data.

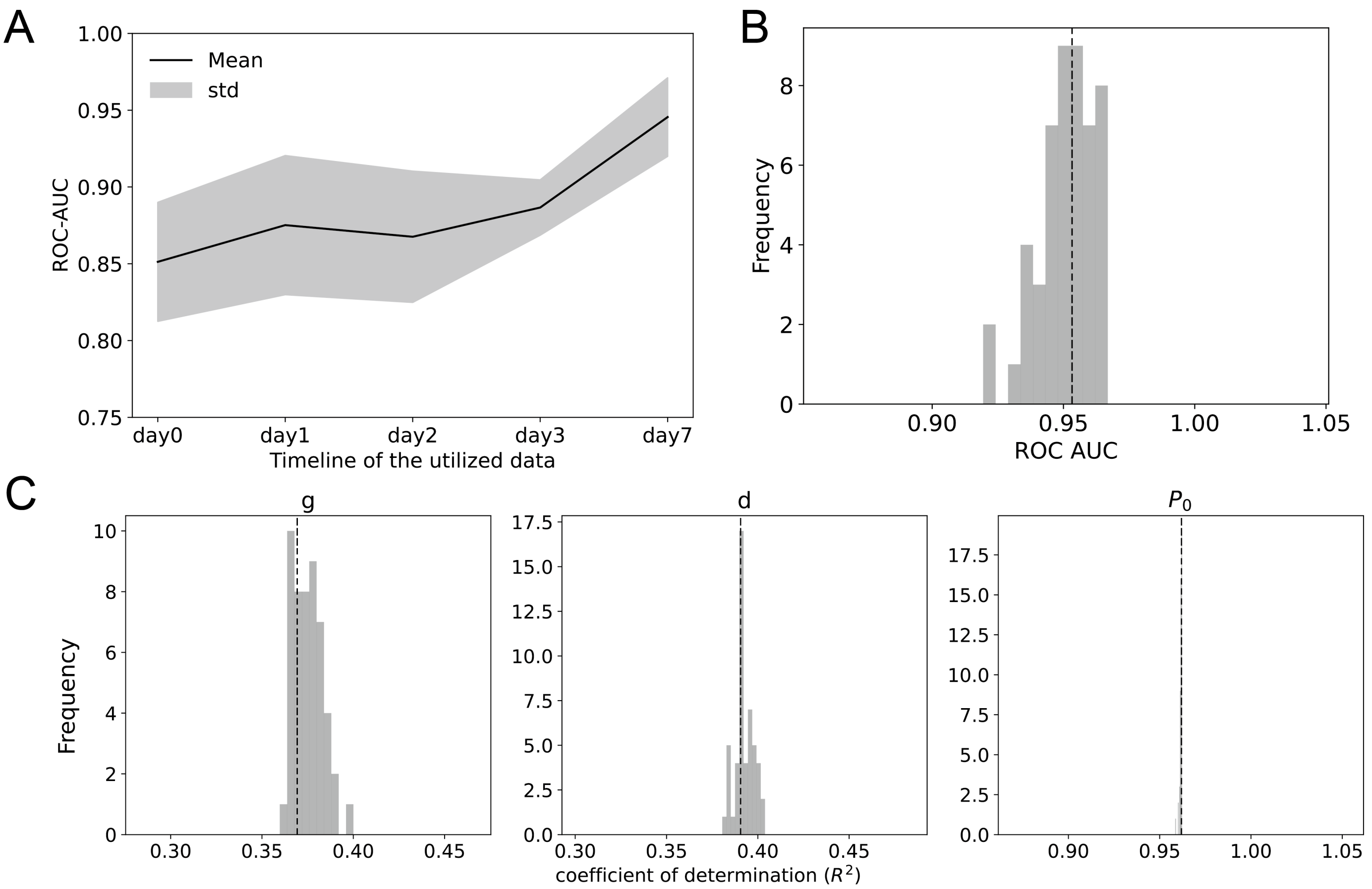

Supplementary Figure 2 | Sensitivity analysis of RF on different datasets: (A) The ROC-AUCs of RF classifiers trained to predict the need for transplantation based on blood test data measured at different dates after admission are displayed (using data from **Fig S1B**). The mean values (black line) and standard deviations (gray shaded area) were plotted using k-fold cross-validation. **(B)** The distribution of ROC-AUCs of the RF classifiers based on 50 different datasets with missing values imputed using multiple imputation on blood test data at 7 days post-admission is depicted (using data from **Fig S1A**). The average and standard deviation of ROC-AUC is 0.95 ± 0.01 and the vertical dashed line is the corresponding ROC-AUC in **Fig 1A**. **(C)** The distributions of the coefficient of determination (i.e., R^2) between the predicted parameters of g, d, and P_0 by the RF prediction on admission (using data from **Fig S1C**) and the estimated parameters by the model fitting are presented (i.e., $R^2 = 0.37 \pm 0.01,\ 0.39 \pm 0.01,\ 0.96 \pm 0.00$, respectively) across the 50 different datasets. The vertical dashed lines are the corresponding R^2 in **Fig S6**.

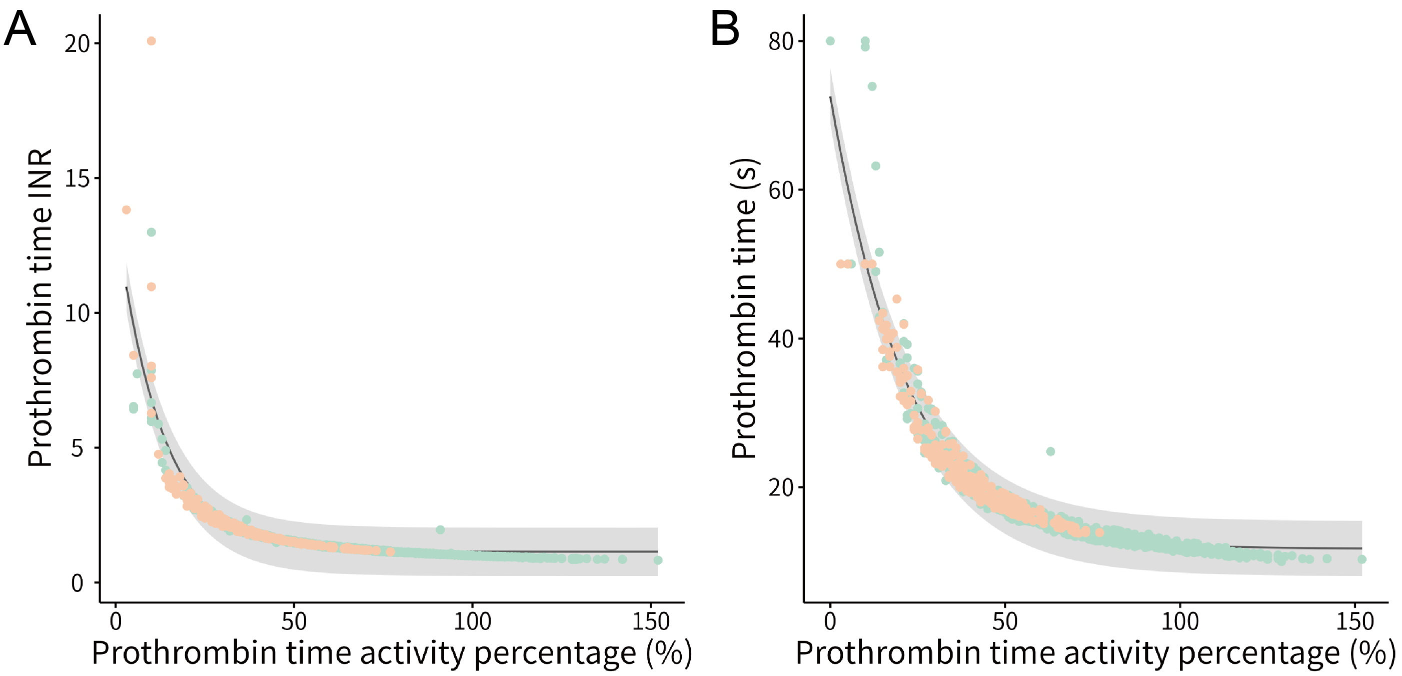

Supplementary Figure 3 | Correlations between PT% and other PT metrics among ALI patients: The correlations between PT% and PT-INR, as well as between PT% and Prothrombin time (seconds) (i.e. PTs), from the same samples, fitted with $(\mathrm{INR}) = 1.14 + 955.20/(1 + \exp(0.08(\mathrm{PT\%}) + 54.16)$ and $(\mathrm{PTs}) = 11.75 + 618.41/(1 + \exp(0.05(\mathrm{PT\%}) + 50.00)$, are illustrated in **(A)** and **(B)**, respectively. Each data point, colored accordingly, and the black curve represent an individual sample and the model fitting, with the correlations 0.96 and 0.89 calculated as Mutual Information Coefficient (MIC), respectively.

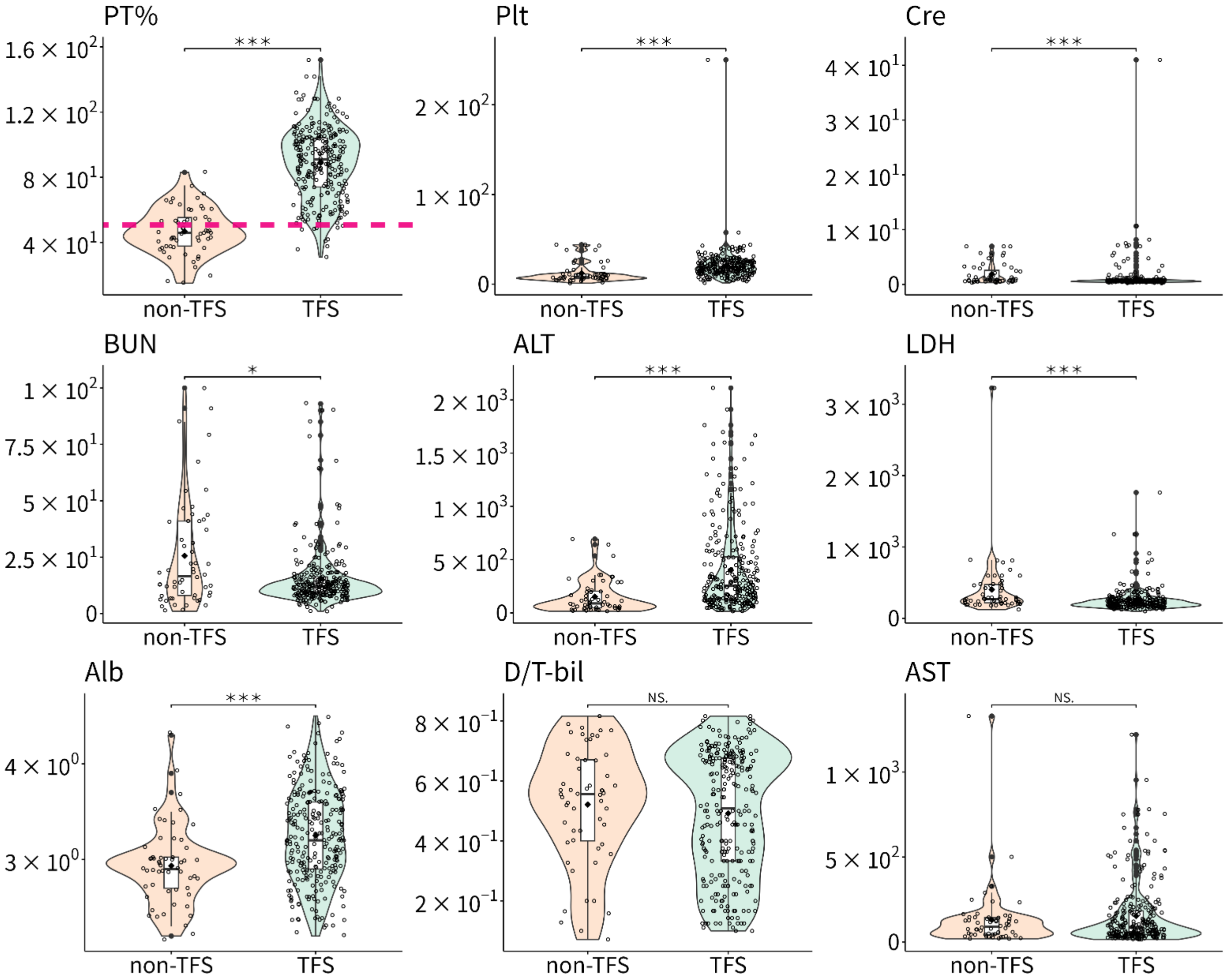

Supplementary Figure 4 | Comparison of blood test data between TFS and non-TFS patients: The blood test data on day 7 in **Fig 1B** are plotted and colored accordingly. Statistical significance was calculated by using the Wilcoxon rank-sum test (NS.: p-value > 0.05, *: p-value ≤ 0.05, **: p-value ≤ 0.01, and ***: p-value ≤ 0.001, respectively). The red dashed line in the first panel indicates the PT% threshold value (i.e., PT%=51.30%).

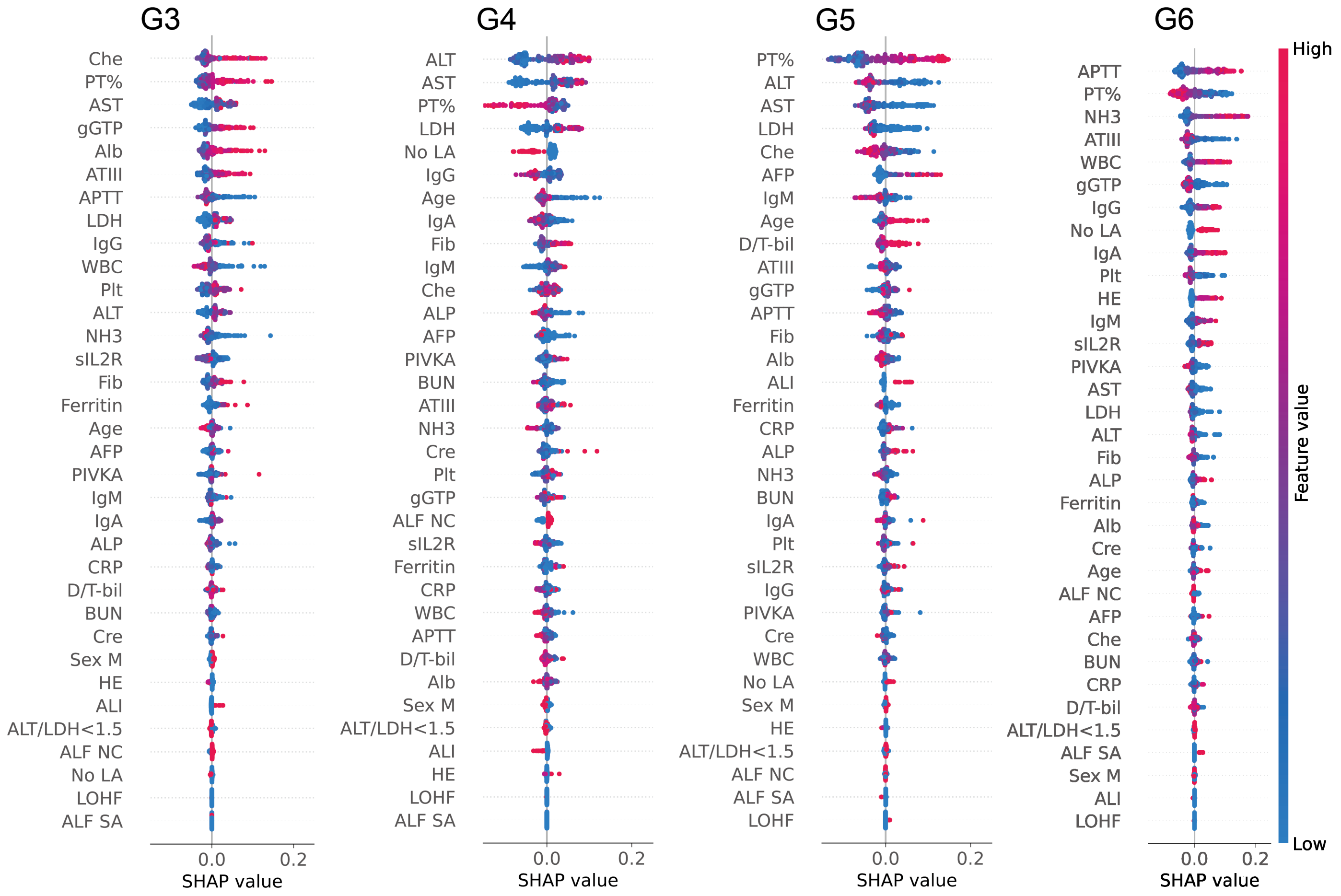

Supplementary Figure 5 | Comparison of admission test data among stratified groups: All features of importance in the predictive model in **Fig 2C** are illustrated as a SHAP summary plot (using data from **Fig S1C**).

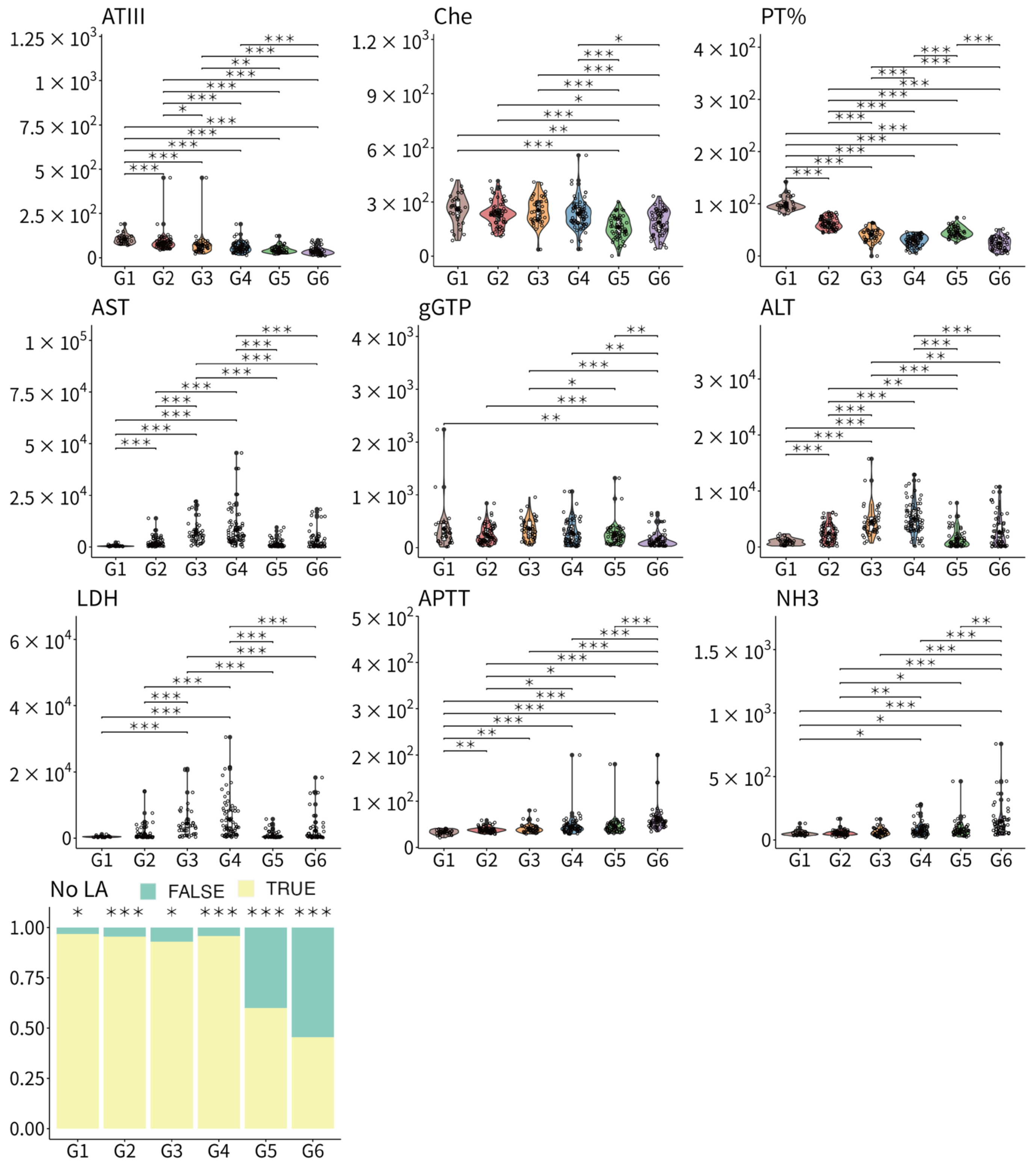

Supplementary Figure 6 | Comparison of blood test data among stratified groups: The blood test data selected as important features by SHAP for G1 to G6 in **Fig 2D** are plotted and colored accordingly. Statistical significance was calculated by using the Wilcoxon rank-sum test for continuous values and p-values were corrected by Bonferroni's method (NS.: p-value > 0.05, *: p-value ≤ 0.05, **: p-value ≤ 0.01, and ***: p-value ≤ 0.001, respectively). Also, Fisher's exact test for categorical values was used, and whether there were significant differences in the proportions of each group relative to the total was examined by applying residual analysis (NS.: p-value > 0.05, *: p-value ≤ 0.05, **: p-value ≤ 0.01, and ***: p-value ≤ 0.001, respectively). "TRUE" indicates that the

item is applicable. For example, in the context of No LA, "TRUE" means cases in which liver atrophy was not observed, and "FALSE" means cases in which liver atrophy was observed.

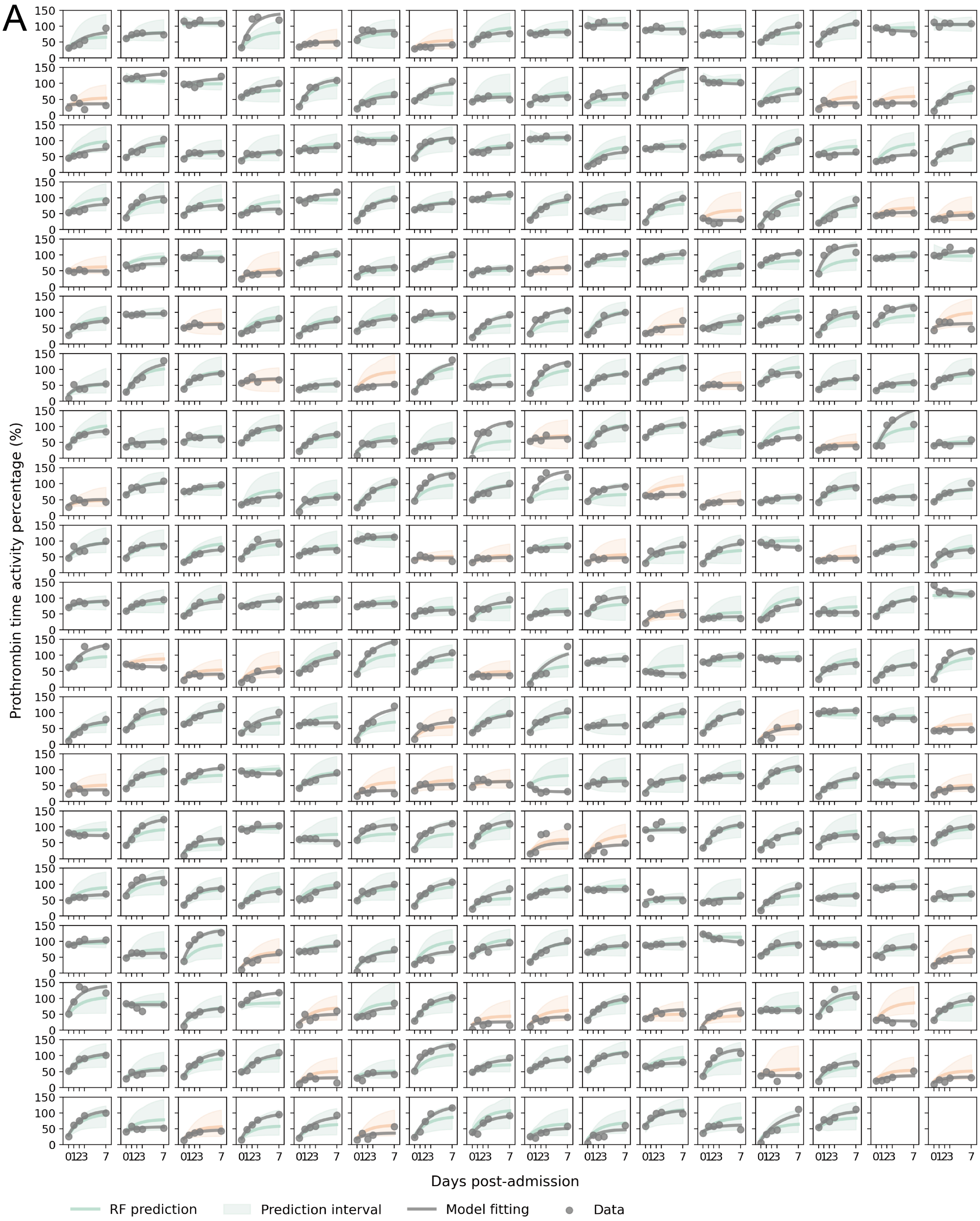
A
Prothrombin time activity percentage (%)
Days post-admission
RF prediction
Prediction interval
Model fitting
Data

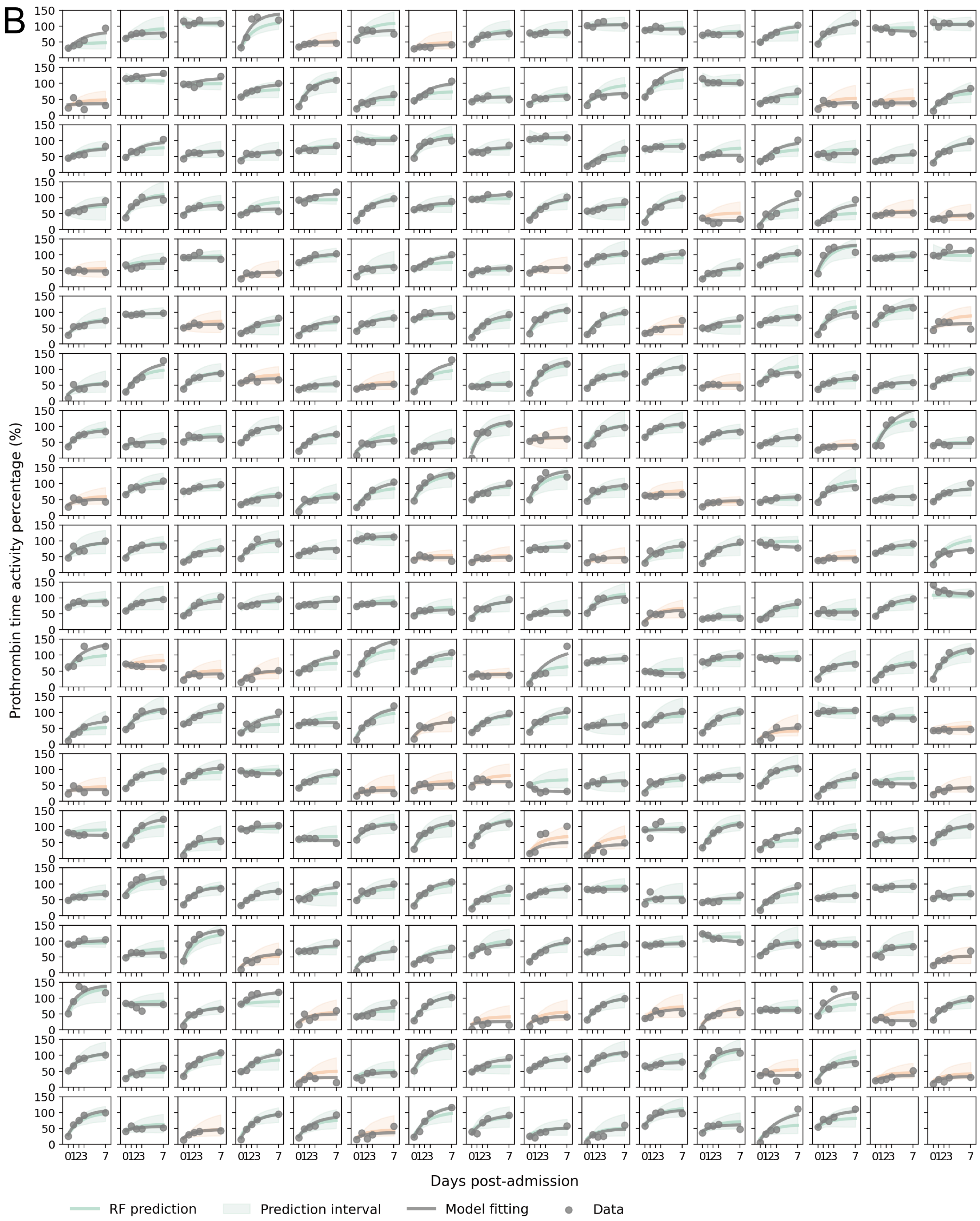

Supplementary Figure 7 | Predicted PT% dynamics along with model fitting: The predicted PT% dynamics by the mathematical model with predicted parameters by RF prediction based on the blood test data at admission and until 2 days post-admission for all patients are presented in **(A)** and **(B)**, respectively (using data from **Fig S1C** and **Fig S1D**).

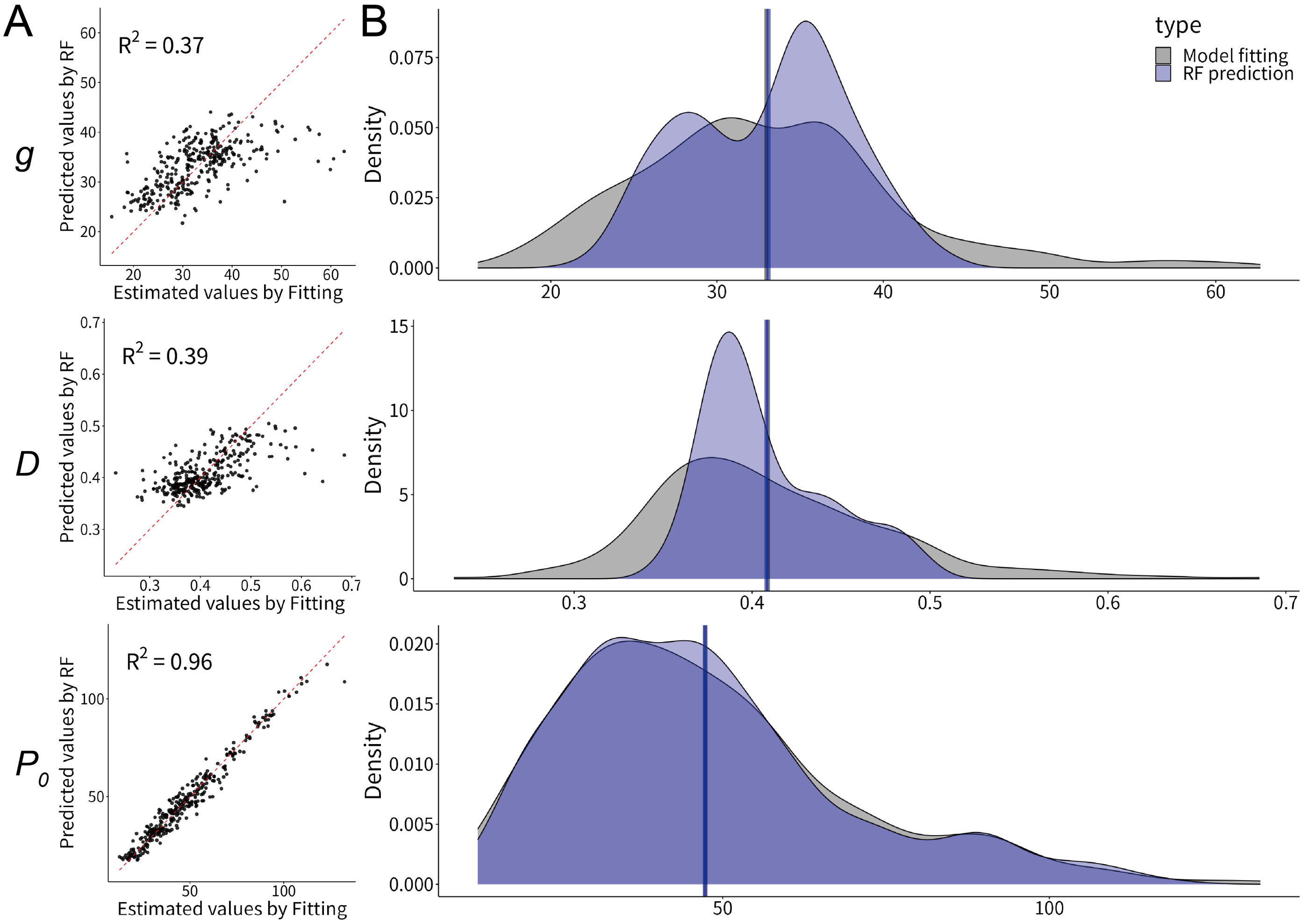

Supplementary Figure 8 | Estimated and predicted parameter values by model fitting and RF prediction: (A) The comparison between the predicted parameters of g, d, and P_0 by the RF prediction (using data from **Fig S1C**) and the estimated parameters by the model fitting with NLMEM (using data from **Fig S1B**) are depicted, respectively. **(B)** The corresponding distributions and mean values of the predicted (using data from **Fig S1C**) and estimated (using data from **Fig S1B**) parameter values are shown in blue and black, respectively.

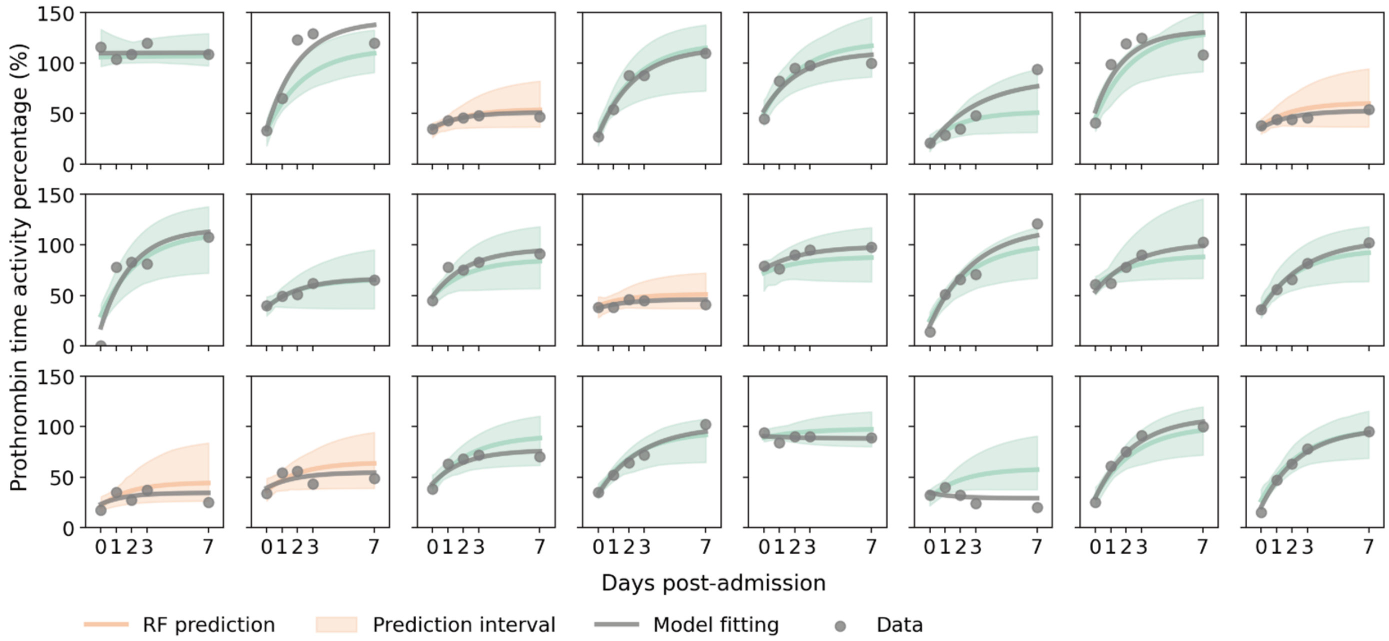

Supplementary Figure 9 | Predicted PT% dynamics with blood test data until 2 days post-admission: The predicted PT% dynamics by the mathematical model with predicted parameters by RF prediction based on the blood test data until 2 days post-admission for all patients are presented. The colored solid line and shaded area in each panel indicate the mean and 95% prediction interval of model prediction, respectively (using data from **Fig S1D**).

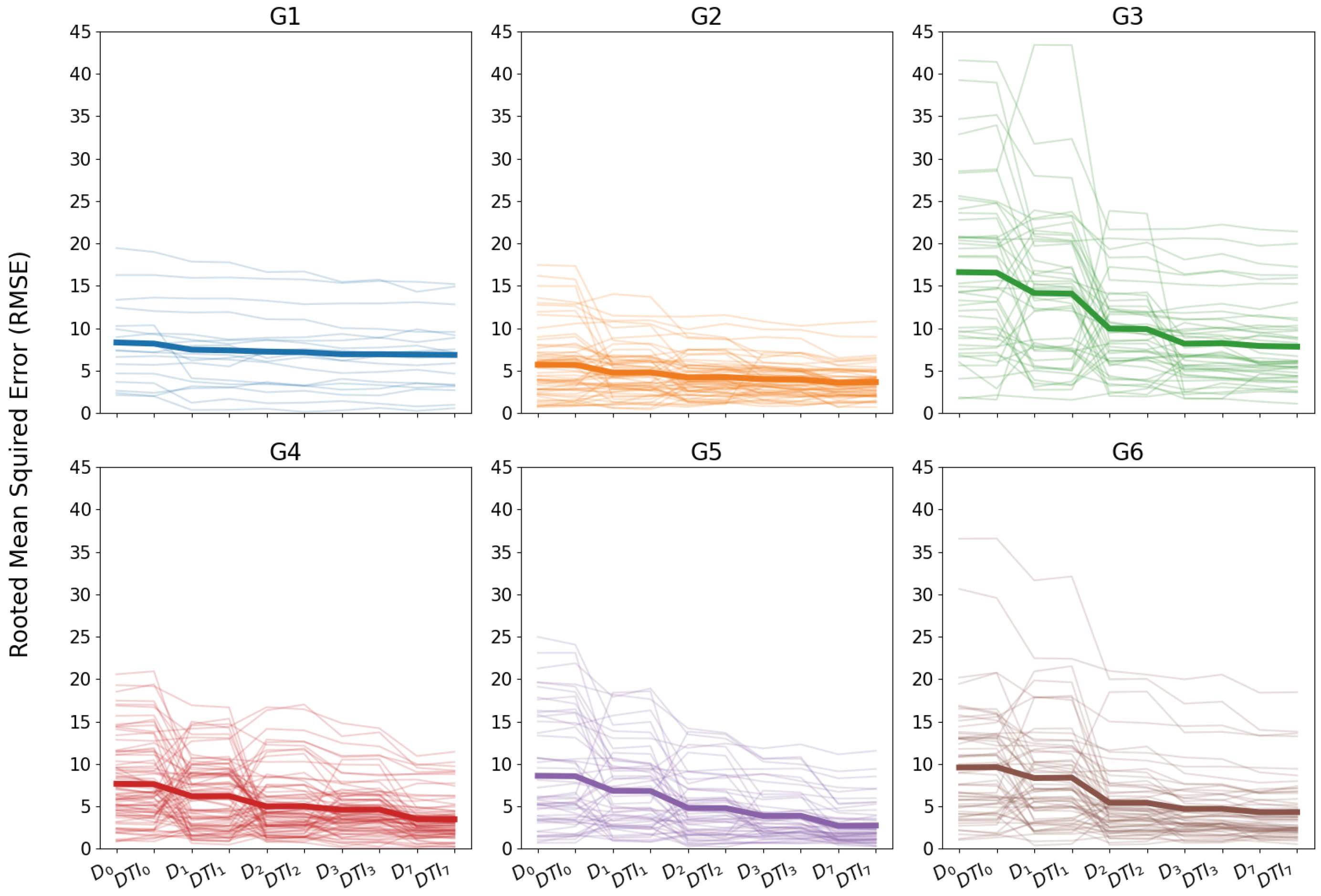

Supplementary Figure 10 | Comparison of RMSEs among the stratified groups: The average and individual RMSEs between the observed PT% data and the RF prediction with different datasets for each group are described in the solid and thin curves, respectively (using data from **Fig S1E**).

Supplementary Table 1 | Clinical numeric data of cohorts from Kyushu University Hospital

Clinical Data	G1 (N=31)	G2 (N=66)	G3 (N=42)	G4 (N=70)	G5 (N=55)	G6 (N=55)	All	p-value
**Basic information**								
Age	48.7 (12.1)	42.2 (16.7)	43.5 (11.6)	44.0 (14.9)	52.5 (14.6)	50.3 (14.9)	46.5 (15.0)	***
**Blood test Data**								
**Day0**								
WBC	6517.4 (2472.7)	5772.6 (3447.5)	4818.7 (3353.0)	6455.2 (4657.1)	8222.2 (5647.1)	10291.6 (6379.8)	7070.7 (4964.4)	***
Plt	21.3 (7.2)	19.8 (20.5)	15.4 (5.3)	15.1 (10.1)	14.6 (7.3)	12.6 (7.3)	16.2 (12.0)	**
Alb	3.8 (0.5)	3.7 (0.4)	3.8 (0.6)	3.6 (0.5)	3.2 (0.6)	3.2 (0.6)	3.5 (0.6)	***
Tbil	2.8 (3.5)	7.3 (6.3)	3.9 (2.4)	4.4 (3.7)	12.1 (9.3)	13.0 (7.7)	7.6 (7.2)	***
Dbil	1.7 (2.7)	5.1 (4.6)	2.5 (1.7)	2.8 (2.6)	8.4 (7.1)	8.4 (5.6)	5.1 (5.3)	***
AST	567.0 (402.3)	2015.4 (2221.0)	6956.9 (5294.1)	8265.9 (8066.9)	1535.8 (2018.6)	3293.8 (4682.0)	4034.6 (5606.6)	***
ALT	938.1 (577.8)	2442.9 (1642.9)	4520.5 (2820.9)	5135.5 (2797.0)	1480.9 (1674.4)	2618.7 (2793.5)	3025.5 (2705.5)	***
LDH	463.6 (225.8)	1390.6 (2188.4)	4541.9 (4812.3)	5681.9 (6079.3)	961.1 (1164.5)	2340.9 (4034.5)	2746.9 (4333.0)	***
ALP	552.1 (370.3)	542.5 (329.8)	427.7 (172.3)	451.9 (217.1)	562.4 (317.4)	575.3 (337.2)	517.5 (298.1)	*
gGTP	356.1 (412.5)	256.6 (176.9)	353.3 (207.1)	288.1 (235.1)	247.1 (205.0)	151.8 (145.7)	266.2 (234.4)	***
Che	257.7 (82.7)	229.3 (62.9)	253.9 (73.4)	230.7 (79.5)	164.4 (69.6)	184.9 (70.4)	216.8 (79.1)	***
NH3	55.6 (22.6)	56.0 (20.9)	62.5 (28.7)	77.2 (42.4)	80.1 (62.6)	146.7 (128.0)	81.3 (71.2)	***
BUN	15.2 (10.9)	12.2 (9.2)	14.1 (9.2)	17.3 (15.3)	19.1 (16.1)	20.4 (18.0)	16.4 (14.1)	*
Cre	1.0 (1.4)	0.7 (0.7)	1.0 (0.7)	1.4 (1.7)	1.0 (0.7)	1.5 (1.5)	1.1 (1.3)	**
CRP	0.5 (0.6)	1.4 (1.8)	2.1 (2.8)	3.3 (7.9)	2.2 (2.6)	2.3 (2.8)	2.1 (4.3)	*
Ferritin	2368.3 (4763.5)	5277.3 (9007.5)	38262.9 (56224.7)	35418.0 (48804.7)	6026.4 (8064.1)	19389.9 (39190.2)	18513.9 (37660.0)	***
PTs	12.1 (0.8)	15.6 (1.9)	23.1 (10.6)	29.1 (10.7)	19.8 (3.4)	35.5 (14.1)	23.4 (11.6)	***
PT%	99.0 (12.9)	62.5 (10.8)	40.5 (13.2)	29.4 (10.1)	45.6 (9.6)	23.8 (11.6)	46.3 (24.6)	***
PTINR	1.0 (0.1)	1.3 (0.1)	1.9 (0.5)	2.7 (1.3)	1.7 (0.3)	4.1 (3.5)	2.2 (1.9)	***
APTT	32.8 (5.3)	39.1 (5.9)	40.7 (10.1)	45.8 (20.9)	46.2 (20.0)	59.8 (25.1)	45.0 (18.8)	***
Fib	270.1 (75.7)	227.6 (69.4)	215.8 (83.1)	208.3 (91.4)	175.1 (86.4)	146.2 (62.1)	202.9 (86.2)	***
ATIII	110.7 (69.1)	82.7 (50.3)	59.3 (20.0)	54.6 (23.5)	43.5 (18.5)	35.3 (19.2)	61.2 (42.1)	***
IgG	1285.5 (514.1)	1369.7 (522.8)	1237.2 (825.3)	1207.7 (436.6)	1646.7 (1001.6)	1709.7 (682.9)	1414.9 (706.8)	*
IgA	234.3 (106.6)	258.1 (107.0)	273.6 (87.3)	265.8 (129.0)	312.9 (131.2)	375.7 (195.6)	289.2 (139.5)	***
IgM	152.4 (113.9)	172.9 (127.3)	130.5 (89.4)	169.2 (120.4)	149.4 (131.1)	223.2 (200.5)	169.1 (138.8)	N.S.
IgE	383.9 (1000.9)	419.4 (820.1)	740.8 (1699.9)	503.5 (898.2)	474.0 (888.7)	601.5 (1002.6)	517.5 (1047.1)	*
AFP	5.8 (4.7)	77.4 (428.0)	8.2 (17.6)	7.2 (15.3)	89.3 (212.6)	35.9 (101.8)	40.8 (219.5)	N.S.
PIVKA	161.2 (464.1)	68.4 (178.5)	3260.9 (18288.4)	74.8 (199.9)	417.6 (2365.8)	36.1 (25.9)	553.8 (6726.1)	N.S.
sIL2R	1523.3 (1109.9)	1872.3 (1628.4)	1445.1 (1089.7)	1777.4 (1277.2)	2255.9 (2264.5)	2696.1 (2165.5)	1969.5 (1733.1)	**
D-bil/T-bil	0.4 (0.2)	0.6 (0.1)	0.6 (0.1)	0.6 (0.1)	0.6 (0.1)	0.6 (0.1)	0.6 (0.2)	***
MELDscore	6.0 (6.3)	11.7 (5.1)	17.0 (8.6)	20.9 (10.0)	18.2 (7.1)	29.1 (11.4)	18.0 (10.8)	***
**Day1**								
Plt	21.5 (7.3)	17.1 (8.4)	14.0 (5.2)	12.4 (5.7)	12.2 (6.3)	12.3 (13.7)	14.4 (8.8)	***
Alb	3.4 (0.5)	3.5 (0.4)	3.5 (0.4)	3.4 (0.4)	3.1 (0.5)	3.1 (0.4)	3.3 (0.4)	***
Tbil	2.7 (3.3)	6.2 (5.4)	3.6 (1.6)	4.1 (2.5)	9.7 (7.5)	10.3 (6.0)	6.4 (5.7)	***
Dbil	1.6 (2.5)	4.2 (4.0)	2.1 (1.2)	2.4 (1.7)	6.5 (5.9)	6.0 (4.3)	4.0 (4.1)	***
AST	449.7 (301.6)	878.8 (589.3)	1955.1 (2143.7)	2591.7 (3399.6)	814.2 (1262.9)	1194.6 (2159.8)	1398.0 (2192.5)	***
ALT	781.8 (477.9)	1397.1 (787.7)	1628.2 (1163.7)	1798.8 (1177.3)	776.1 (1097.2)	929.6 (1038.4)	1268.2 (1085.6)	***
LDH	384.0 (217.4)	524.9 (453.7)	919.4 (1100.0)	1332.4 (1971.5)	630.6 (1034.8)	924.7 (1887.1)	827.5 (1391.5)	**
NH3	66.4 (33.8)	68.1 (28.0)	70.2 (22.3)	82.1 (42.6)	79.9 (28.6)	127.4 (63.7)	83.6 (44.6)	***

BUN	15.3 (12.3)	11.7 (9.3)	11.4 (7.5)	15.4 (14.2)	17.1 (16.4)	16.2 (14.1)	14.6 (12.9)	N.S.
Cre	1.0 (1.6)	0.7 (0.7)	0.9 (0.6)	1.3 (1.6)	0.9 (0.7)	1.3 (1.4)	1.0 (1.2)	*
PTs	12.6 (1.6)	14.2 (1.3)	14.9 (2.1)	18.0 (3.1)	16.4 (2.1)	21.6 (4.6)	16.6 (3.9)	***
PT%	94.5 (11.7)	72.6 (9.4)	68.8 (14.8)	50.4 (10.8)	57.7 (9.1)	39.6 (10.0)	61.1 (19.2)	***
PTINR	1.1 (0.2)	1.2 (0.1)	1.3 (0.2)	1.6 (0.3)	1.4 (0.2)	1.9 (0.4)	1.4 (0.4)	***
D-bil/T-bil	0.4 (0.2)	0.6 (0.1)	0.6 (0.1)	0.6 (0.1)	0.6 (0.1)	0.6 (0.1)	0.6 (0.2)	
**Day2**								***
Plt	21.6 (7.8)	18.3 (9.1)	13.4 (4.8)	12.6 (5.4)	12.4 (6.1)	10.0 (5.2)	14.3 (7.5)	***
Alb	3.4 (0.5)	3.4 (0.4)	3.3 (0.3)	3.3 (0.3)	3.0 (0.4)	3.1 (0.4)	3.2 (0.4)	***
Tbil	2.6 (3.7)	4.9 (4.3)	3.1 (2.2)	3.6 (1.8)	9.5 (8.3)	10.5 (5.8)	5.9 (5.8)	***
Dbil	1.6 (2.8)	3.3 (3.1)	1.8 (1.6)	2.1 (1.3)	6.4 (6.4)	5.8 (4.2)	3.7 (4.1)	***
AST	351.7 (276.2)	477.7 (441.3)	454.3 (430.7)	587.3 (566.9)	424.7 (533.7)	368.5 (435.1)	458.5 (477.1)	N.S.
ALT	688.0 (441.7)	1028.2 (628.6)	889.6 (627.8)	858.3 (580.6)	502.1 (680.3)	425.3 (419.3)	745.0 (618.0)	***
LDH	346.7 (170.1)	356.2 (313.3)	281.5 (181.4)	339.5 (177.5)	376.0 (359.6)	426.6 (336.2)	357.4 (277.0)	N.S.
NH3	64.5 (27.5)	71.2 (24.6)	67.2 (19.5)	85.1 (41.1)	85.3 (34.6)	133.4 (56.3)	86.2 (43.3)	***
BUN	15.1 (12.6)	12.7 (9.6)	11.9 (6.9)	15.6 (14.6)	16.7 (16.2)	14.5 (13.6)	14.5 (12.8)	N.S.
Cre	1.1 (1.8)	0.7 (0.7)	0.8 (0.6)	1.2 (1.5)	0.8 (0.7)	1.5 (1.6)	1.0 (1.2)	**
PTs	12.4 (1.5)	13.5 (1.2)	13.2 (1.8)	16.3 (2.6)	16.3 (1.8)	23.0 (6.2)	16.1 (4.6)	***
PT%	100.9 (10.2)	78.8 (10.8)	87.0 (20.1)	58.8 (12.3)	58.0 (8.3)	37.5 (10.8)	66.9 (22.9)	***
PTINR	1.0 (0.1)	1.1 (0.1)	1.1 (0.2)	1.4 (0.2)	1.4 (0.1)	2.0 (0.5)	1.4 (0.4)	***
D-bil/T-bil	0.4 (0.2)	0.8 (1.7)	0.5 (0.1)	0.6 (0.1)	0.6 (0.2)	0.5 (0.1)	0.6 (0.8)	N.S.
**Day3**								
Plt	22.1 (8.0)	22.4 (25.9)	13.5 (5.7)	13.9 (10.8)	12.9 (7.2)	9.6 (5.0)	15.5 (14.4)	***
Alb	3.4 (0.4)	3.3 (0.4)	3.1 (0.4)	3.2 (0.4)	3.0 (0.4)	3.2 (0.5)	3.2 (0.4)	***
Tbil	2.7 (4.0)	4.6 (4.0)	2.7 (2.6)	3.2 (1.9)	9.0 (8.5)	10.6 (6.1)	5.7 (5.9)	***
Dbil	1.6 (2.9)	2.9 (2.8)	1.8 (2.0)	1.9 (1.3)	6.1 (6.7)	6.0 (5.0)	3.5 (4.3)	***
AST	287.1 (246.3)	392.2 (403.9)	213.4 (202.8)	278.6 (239.0)	281.5 (277.5)	218.8 (220.2)	284.5 (286.2)	**
ALT	584.3 (411.3)	926.0 (608.5)	677.8 (460.3)	640.4 (497.9)	404.4 (505.7)	303.9 (273.3)	600.3 (523.3)	***
LDH	311.9 (154.6)	329.1 (228.5)	230.3 (84.8)	277.2 (127.9)	326.0 (256.1)	357.6 (218.6)	307.4 (195.6)	*
BUN	16.7 (15.0)	13.5 (8.5)	13.7 (8.7)	17.1 (14.1)	17.1 (16.4)	16.0 (15.8)	15.7 (13.4)	N.S.
Cre	1.1 (1.9)	0.7 (0.6)	1.0 (1.0)	1.3 (1.7)	0.8 (0.7)	1.5 (1.7)	1.1 (1.3)	**
PTs	12.3 (1.1)	13.1 (1.0)	12.4 (2.0)	14.9 (2.0)	16.2 (1.6)	22.0 (6.0)	15.4 (4.4)	***
PT%	102.9 (12.1)	83.1 (10.9)	99.1 (20.8)	67.8 (14.1)	58.9 (7.5)	39.5 (11.0)	72.1 (24.5)	***
PTINR	1.0 (0.1)	1.1 (0.1)	1.0 (0.2)	1.3 (0.2)	1.4 (0.1)	1.9 (0.5)	1.3 (0.4)	***
D-bil/T-bil	0.4 (0.2)	0.6 (0.2)	0.8 (1.6)	0.6 (0.1)	0.6 (0.2)	0.5 (0.2)	0.6 (0.6)	N.S.
**Day7**								
Plt	31.8 (41.3)	23.4 (10.3)	20.2 (7.5)	17.0 (7.2)	13.5 (7.9)	8.9 (4.8)	18.2 (16.1)	***
Alb	3.5 (0.5)	3.4 (0.4)	3.4 (0.5)	3.1 (0.5)	2.9 (0.4)	3.1 (0.4)	3.2 (0.5)	***
Tbil	2.6 (5.5)	4.4 (3.8)	3.1 (5.5)	3.6 (3.1)	10.0 (7.9)	11.9 (6.5)	11.7 (6.8)	***
Dbil	1.6 (4.5)	2.7 (2.9)	1.3 (1.9)	2.4 (2.4)	6.7 (6.2)	6.6 (5.0)	6.5 (5.1)	***
AST	173.2 (208.3)	218.6 (209.3)	98.2 (128.2)	94.0 (113.1)	204.1 (205.0)	103.5 (177.0)	115.6 (179.2)	***
ALT	335.3 (335.9)	597.0 (436.4)	349.8 (334.7)	338.3 (317.2)	354.3 (449.6)	129.1 (145.1)	134.5 (137.3)	***
LDH	262.7 (152.2)	253.8 (112.1)	205.7 (57.6)	227.5 (132.2)	321.5 (249.1)	350.6 (413.4)	381.8 (427.9)	**
BUN	17.0 (15.4)	12.8 (5.0)	13.4 (8.3)	19.3 (20.0)	17.1 (11.1)	21.3 (23.1)	16.9 (15.6)	**
Cre	1.1 (1.8)	0.6 (0.2)	2.1 (6.3)	1.3 (1.7)	0.8 (0.6)	1.7 (2.0)	1.2 (2.7)	*
PTs	12.0 (1.7)	12.4 (0.6)	11.9 (3.0)	12.7 (1.2)	16.5 (2.0)	20.8 (5.4)	14.5 (4.3)	***
PT%	102.3 (13.2)	91.7 (9.7)	113.1 (14.2)	88.9 (13.1)	58.2 (7.3)	46.1 (15.5)	81.3 (25.8)	***
PTINR	1.0 (0.2)	1.1 (0.1)	1.0 (0.2)	1.1 (0.1)	1.4 (0.1)	1.8 (0.5)	1.2 (0.4)	***
D-bil/T-bil	0.3 (0.2)	0.5 (0.2)	0.4 (0.2)	0.5 (0.2)	0.6 (0.2)	0.5 (0.2)	0.5 (0.2)	***

**Other information**								
Encephalopathy (grade)								
Day0: On Admission	0.0 (0.0)	0.0 (0.2)	0.1 (0.3)	0.3 (0.7)	0.4 (0.9)	1.2 (1.5)	0.4 (0.9)	***
Day7: Worst for 7 days	0.1 (0.4)	0.0 (0.2)	0.1 (0.3)	0.3 (0.7)	0.6 (1.2)	1.6 (1.5)	0.5 (1.0)	***
**Treatment information**								
FFP prescribing								
Day0	0.0 (0.0)	11.8 (16.7)	27.0 (16.2)	30.4 (12.7)	21.2 (16.3)	30.5 (13.6)	21.6 (17.4)	***
Day1	0.0 (0.0)	9.3 (16.1)	17.3 (19.4)	29.7 (14.9)	17.3 (16.5)	32.3 (16.9)	19.3 (18.9)	***
Day2	0.0 (0.0)	2.5 (9.6)	6.7 (15.1)	20.2 (18.0)	14.6 (17.0)	29.8 (18.2)	13.5 (18.1)	***
Day3	0.0 (0.0)	0.3 (2.5)	0.0 (0.0)	14.0 (30.1)	25.2 (46.9)	79.2 (60.6)	21.1 (44.5)	***

†：mean (standard deviation)

‡：ANOVA, *: p<0.05, **: p<0.01, ***: p<0.001

Supplementary Table 2 | Clinical categorical data of cohorts from Kyushu University Hospital

Clinical Data	G1 (N=31)	G2 (N=66)	G3 (N=42)	G4 (N=70)	G5 (N=55)	G6 (N=55)	All	p-value
**Clinical outcomes**								***
TFS	100.0 (31)	100.0 (66)	100.0 (42)	97.1 (68)	76.4 (42)	27.3 (15)	82.5 (264)	
non-TFS	0.0 (0)	0.0 (0)	0.0 (0)	2.9 (2)	23.6 (13)	72.7 (40)	17.2 (55)	
**Basic information**								
Sex								*
M	29.0 (9)	59.1 (39)	66.7 (28)	50.0 (35)	54.5 (30)	47.3 (26)	52.2 (167)	
F	71.0 (22)	40.9 (27)	33.3 (14)	50.0 (35)	45.5 (25)	52.7 (29)	47.5 (152)	
**Admission data**								
diagnosis								***
ALI	100.0 (31)	77.3 (51)	11.9 (5)	0.0 (0)	21.8 (12)	0.0 (0)	30.9 (99)	
ALF without coma	0.0 (0)	22.7 (15)	85.7 (36)	87.1 (61)	60.0 (33)	43.6 (24)	52.8 (169)	
ALF with coma (acute)	0.0 (0)	0.0 (0)	2.4 (1)	12.9 (9)	12.7 (7)	47.3 (26)	13.4 (43)	
ALF with coma (subacute)	0.0 (0)	0.0 (0)	0.0 (0)	0.0 (0)	0.0 (0)	5.5 (3)	0.9 (3)	
LOHF	0.0 (0)	0.0 (0)	0.0 (0)	0.0 (0)	5.5 (3)	3.6 (2)	1.6 (5)	
LiverAtrophy +	3.2 (1)	4.5 (3)	7.1 (3)	2.9 (2)	40.0 (22)	52.7 (29)	18.8 (60)	***
ALT/LDH < 1.5	9.7 (3)	21.2 (14)	57.1 (24)	54.3 (38)	18.2 (10)	21.8 (12)	31.6 (101)	***
**Admission Treatment**								
**Day0**								
PE	0.0 (0)	33.3 (22)	71.4 (30)	87.1 (61)	65.5 (36)	96.4 (53)	63.1 (202)	***
Steroid pulse therapy	41.9 (13)	74.2 (49)	73.8 (31)	71.4 (50)	76.4 (42)	63.6 (35)	68.8 (220)	***
TASIT	0.0 (0)	16.7 (11)	61.9 (26)	48.6 (34)	25.5 (14)	32.7 (18)	32.2 (103)	***
peripheral pulse	41.9 (13)	62.1 (41)	16.7 (7)	25.7 (18)	56.4 (31)	32.7 (18)	40.0 (128)	***
Anticoagulation	0.0 (0)	31.8 (21)	61.9 (26)	78.6 (55)	61.8 (34)	65.5 (36)	53.8 (172)	***
CHDF	0.0 (0)	0.0 (0)	2.4 (1)	18.6 (13)	16.4 (9)	45.5 (25)	15.0 (48)	***
Vasopressor Use	0.0 (0)	1.5 (1)	0.0 (0)	1.4 (1)	3.6 (2)	5.5 (3)	2.2 (7)	N.S.
**Other information (utilized as day7 data)**								
Etiology								***
HAV	0.0 (0)	19.7 (13)	11.9 (5)	17.1 (12)	10.9 (6)	3.6 (2)	11.9 (38)	
HBV	16.1 (5)	27.3 (18)	16.7 (7)	24.3 (17)	20.0 (11)	43.6 (24)	25.6 (82)	
AIH	32.3 (10)	21.2 (14)	0.0 (0)	1.4 (1)	25.5 (14)	12.7 (7)	14.4 (46)	
DILI	16.1 (5)	4.5 (3)	0.0 (0)	5.7 (4)	5.5 (3)	0.0 (0)	4.7 (15)	
Others	19.4 (6)	10.6 (7)	16.7 (7)	14.3 (10)	16.4 (9)	12.7 (7)	14.4 (46)	
unknown	16.1 (5)	16.7 (11)	54.8 (23)	37.1 (26)	21.8 (12)	27.3 (15)	28.7 (92)	

†：ratio (count number)

‡：Fisher's exact test,, *: p<0.05, **: p<0.01, ***: p<0.001

Supplementary Table 3| Estimated fixed and random effects for PT% dynamics

Parameters	Description	Unit	$\boldsymbol{\vartheta}$: Fixed effect (SE)*	$\boldsymbol{\Omega}$: SD of random effect (SE)*
g	The rate of increase in PT% due to the consumption and breakdown of coagulation factors	Day^{-1}	31.5 (0.877)	0.29 (0.018)
D	The rate of decrease in PT% due to the production of coagulation factors by the liver	Day^{-1}	0.397 (0.012)	0.29 (0.023)
$P(0)$	Initial PT% value on admission	%	42.1 (1.28)	0.51 (0.025)

*The parameter for patient k, ϑ_i $(= \vartheta \times e^{\pi_k})$, is represented as a product of ϑ (a fixed effect) and e^{π_k} (a random effect).
π_k follows the normal distribution with mean 0 and standard deviation Ω. SE: standard error.

Supplementary Table 4| Comparison of the proposed model with previous models

Predicted outcome	Predictors *	Data description†	Dataset split (train / test)	Evaluation of model	Reference
Disease progression dynamics	Dynamics prediction	319 patients with ALF/ALI (APAP: 2, others: 317)	Leave-one-out	R^{2} : 0.85	This study
Need for liver transplantation.	KCC	763 patients with FHF (APAP: 431, others: 332)	77% : 23%(588 / 175)	No reported	[1]
Transplant-free Survival (TFS)	ALFSGPI	1974 patients with ALF (APAP:933, others:1041)	50% : 50%(987 / 987)	C statistics: 0.84 (in validation data)	[2]
Mortality	ALFED	380 patients with ALF (APAP: 0, others: 380)	64% : 36%(244 / 136)	AUROC: 0.92	[3]
Mortality	LDH	58 patients with ALF+ 10 volunteers (APAP:58, others: 0)	No reported	AUROC: 0.67	[4]
Transplant-free Survival (TFS)	CXCL14	130 patients with ALF (APAP: 130, others: 0)	No reported	AUROC: 0.86	[5]
Transplant-free Survival (TFS)	PT	595 patients with ALF/ALI (APAP: 0, others: 595)	No reported	AUROC: 0.89	[6]
Mortality	MELD	6769 LT waitlist registers	No reported	No reported	[7]

*KCC: King's College Criteria, ALFSGPI: Acute Liver Failure Study Group Prognostic Index, ALFED: Acute Liver Failure Early Dynamic model, LDH: lactate dehydrogenase, CXCL14: plasma chemokine C-X-C motif Ligand 14, PT: Prothrombin time, MELD: Model for End-Stage Liver Disease

†ALF: Acute Liver Failure, ALI: Acute Liver Injury, APAP: Patients whose etiology is acetaminophen, others: Patients whose etiology is not acetaminophen.

REFERENCES

1. O'Grady, J.G., et al., *Early indicators of prognosis in fulminant hepatic failure.* Gastroenterology, 1989. **97**(2): p. 439-45.
2. Koch, D.G., et al., *Development of a Model to Predict Transplant-free Survival of Patients With Acute Liver Failure.* Clin Gastroenterol Hepatol, 2016. **14**(8): p. 1199-1206 e2.
3. Kumar, R., et al., *Prospective derivation and validation of early dynamic model for predicting outcome in patients with acute liver failure.* Gut, 2012. **61**(7): p. 1068-75.
4. Vazquez, J.H., et al., *Proteomics Indicates Lactate Dehydrogenase Is Prognostic in Acetaminophen-Induced Acute Liver Failure Patients and Reveals Altered Signaling Pathways.* Toxicol Sci, 2022. **187**(1): p. 25-34.
5. Umbaugh, D.S., et al., *The chemokine CXCL14 is a novel early prognostic biomarker for poor outcome in acetaminophen-induced acute liver failure.* Hepatology, 2024. **79**(6): p. 1352-1364.
6. Kumagai, K., et al., *Early-phase prothrombin time-international normalized ratio in acute liver injury indicates the timing of therapeutic intervention and predicts prognostic improvement.* Hepatol Res, 2023. **53**(2): p. 160-171.
7. Kim, W.R., et al., *Hyponatremia and mortality among patients on the liver-transplant waiting list.* N Engl J Med, 2008. **359**(10): p. 1018-26.

LIST OF SUPPLEMENTARY MATERIALS

Supplementary Figure 1 | List of datasets utilized in our analysis

Supplementary Figure 2 | Sensitivity analysis of RF on different datasets

Supplementary Figure 3 | Correlations between PT% and PT-INR among ALI patients

Supplementary Figure 4 | Comparison of blood test data between TFS and non-TFS patients

Supplementary Figure 5 | Comparison of admission test data among stratified groups

Supplementary Figure 6 | Comparison of blood test data among stratified groups

Supplementary Figure 7 | Predicted PT% dynamics along with model fitting

Supplementary Figure 8 | Estimated and predicted parameter values by model fitting and RF prediction

Supplementary Figure 9 | Predicted PT% dynamics along with model fitting

Supplementary Figure 10 | Comparison of RMSEs among the stratified groups

Supplementary Table 1 | Clinical numeric data of cohorts from Kyushu University Hospital

Supplementary Table 2 | Clinical categorical data of cohorts from Kyushu University Hospital

Supplementary Table 3 | Estimated fixed and random effects for PT% dynamics

Supplementary Table 4 | Comparison of the proposed model with previous models
